# Quantifying conditioned place preference: a review of current analyses and a proposal for a novel approach

**DOI:** 10.3389/fnbeh.2023.1256764

**Published:** 2023-08-24

**Authors:** Justin R. Yates

**Affiliations:** Department of Psychological Science, Northern Kentucky University, Highland Heights, KY, United States

**Keywords:** conditioned place preference, adjusted CPP score, classification and regression tree analysis, tolerance interval, extinction, reinstatement, conditioned place aversion, replication

## Abstract

Conditioned place preference (CPP) is used to measure the conditioned rewarding effects of a stimulus, including food, drugs, and social interaction. Because various analytic approaches can be used to quantify CPP, this can make direct comparisons across studies difficult. Common methods for analyzing CPP involve comparing the time spent in the CS^+^ compartment (e.g., compartment paired with drug) at posttest to the time spent in the CS^+^ compartment at pretest or to the CS^–^ compartment (e.g., compartment paired with saline) at posttest. Researchers can analyze the time spent in the compartment(s), or they can calculate a difference score [(CS^+^_*post*_ – CS^+^_*pre*_) or (CS^+^_*post*_ – CS^–^_*post*_)] or a preference ratio (e.g., CS^+^_*post*_/(CS^+^_*post*_ + CS^–^_*post*_)). While each analysis yields results that are, overall, highly correlated, there are situations in which different analyses can lead to discrepant interpretations. The current paper discusses some of the limitations associated with current analytic approaches and proposes a novel method for quantifying CPP, the adjusted CPP score, which can help resolve the limitations associated with current approaches. The adjusted CPP score is applied to both hypothetical and previously published data. Another major topic covered in this paper is methodologies for determining if individual subjects have met criteria for CPP. The paper concludes by highlighting ways in which researchers can increase transparency and replicability in CPP studies.

## Introduction

Conditioned place preference (CPP) is used to measure the conditioned rewarding effects of a stimulus, including drugs (see Bardo and Bevins, 2000; Tzschentke, 2007; Bardo et al., 2015; McKendrick and Graziane, 2020), social interaction (Calcagnetti and Schechter, 1992; Ma et al., 2006; Trezza et al., 2009; Yates et al., 2013; Pinheiro et al., 2016; Cann et al., 2020), food (Duarte et al., 2003; Ma et al., 2006; Nesbit et al., 2017; Jamali et al., 2021; Huerta et al., 2022), and copulation (Meerts and Clark, 2007; Ismail et al., 2010; Guterl et al., 2015; Quintana et al., 2019). CPP experiments are primarily conducted in an apparatus composed of either two or three compartments that can vary in one or more ways. The compartments can be painted different colors, can have different types of flooring, and/or can have different scents placed under the flooring. In a typical CPP experiment, subjects are first allowed to explore each compartment of the CPP apparatus. Next, subjects experience several conditioning sessions. During one conditioning session, the subject is isolated to one compartment with the stimulus of interest (CS^+^ compartment). For example, in drug CPP, the subject is injected with a drug like cocaine before being placed in the compartment. During the next conditioning session, the subject is exposed to a compartment that is not paired with the stimulus of interest (CS^–^ compartment). Subjects receive multiple conditioning sessions in alternating fashion. Researchers can perform one conditioning session or two conditioning sessions each day. Finally, subjects are given a posttest, in which they explore each compartment like they did during the pretest.

The CPP paradigm provides researchers an opportunity to study the underlying neurobiology of reward. Subjects can be pretreated with a pharmacological agent such as a receptor agonist/antagonist, a transporter inhibitor, or an enzyme inhibitor before each conditioning session or just prior to the posttest (Kitanaka et al., 2010; Velazquez-Sanchez et al., 2011; Yates et al., 2021a). To further elucidate the neurobiology of reward, specific brain regions can be temporarily inactivated with GABA receptor agonists or can be lesioned (Zhao et al., 2015; Gargiulo et al., 2022). More recently, methods such as optogenetics and chemogenetics have been used to examine the neural circuits that control reward (Chen H. et al., 2021; Weitz et al., 2021).

An important consideration in CPP experiments is that the apparatus can be biased or unbiased. A biased apparatus is one in which subjects spend significantly more time in one compartment relative to another compartment during the pretest. For example, rodents prefer to spend more time in a compartment with a wire mesh floor compared to a compartment with a steel bar floor (Cunningham et al., 2003). In addition to using a biased or an unbiased apparatus, researchers can use a biased or an unbiased experimental design. In a biased design, the compartment paired with the stimulus of interest is determined according to subjects’ pretest scores, such that the stimulus of interest is paired with the initially nonpreferred compartment. In an unbiased design, the CS^+^ compartment is randomized across subjects. Because discussions regarding the use of biased/unbiased CPP chambers and experimental designs have been detailed previously elsewhere (Tzschentke, 1998; Cunningham et al., 2003), the purpose of this paper is to focus more on how CPP is quantified and analyzed. Due to the various analytic approaches one can take when determining if CPP has occurred, this can make replication across studies difficult. After discussing the current methods of analyzing CPP data, I propose a novel way of analyzing CPP data that can be consistently applied across studies to increase replicability and to reduce some of the limitations associated with existing methods (see section “Methods” for specific details).

**FIGURE 1:**
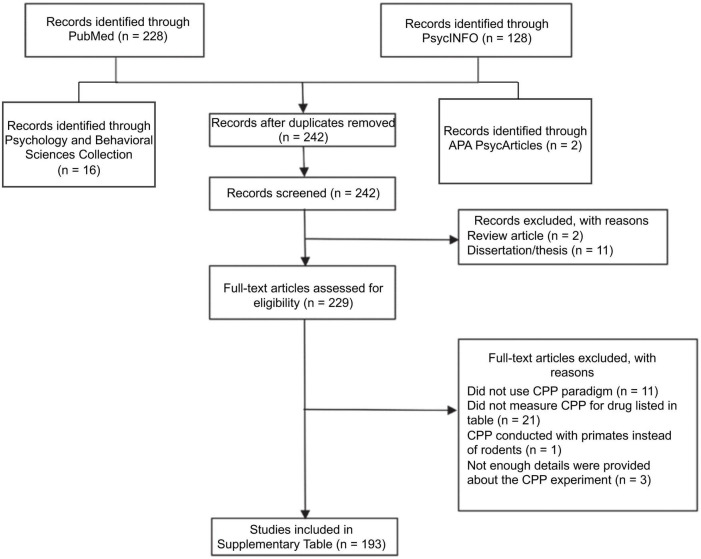
PRISMA (Preferred Reporting Items for Systematic Reviews and Meta-Analyses) flow diagram depicting the literature search for papers published from January 2021 through December 2022 measuring CPP of the stimulants *d*-amphetamine, cocaine, methamphetamine, and nicotine.

Figure 1 shows a flow diagram detailing the literature search used to find recent articles (published from January 2021 through December 2022). The author used the following databases to find articles: PubMed, PsycINFO, Psychology and Behavioral Sciences Collection, and APA PsycArticles. The key terms (conditioned place preference OR place preference) AND (rat OR rats OR mouse OR mice) AND (amphetamine OR cocaine OR methamphetamine OR nicotine) were used. This search yielded 242 unique articles, of which 193 were included in the review. The author first reviewed abstracts of each article to ensure that CPP was being measured. The author reviewed a full-text version of each article to ensure that stimulant CPP was being measured and to determine how the CPP experiment was conducted and how the data were analyzed. Note, some articles have a 2023 date listed, but they were originally published in 2022. As such, they are included in this paper.

Because the purpose of this review was to determine the frequency at which certain analyses are used to assess CPP, I was interested in extracting the following information from the reviewed articles: (1) the drug used in the experiment (e.g., amphetamine), (2) the species being tested (rat vs. mouse), (3) the type of CPP apparatus used (e.g., two compartment vs. three compartment), (4) the design of the experiment (biased vs. unbiased), and (5) the analytic approach used to quantify CPP (see Supplementary Table 1). All 193 studies included in Supplementary Table 1 detailed how CPP was analyzed, although some of the other details were missing or not clearly stated. In Supplementary Table 1, this missing information is denoted by a question mark. There were three studies that did not clearly articulate the methods of the CPP experiment, including how they analyzed CPP data. Therefore, these studies were not included in the review.

CPP experiments are often conducted to compare the conditioned rewarding effects of a stimulus across groups of subjects. For example, CPP can be compared across animals raised in different environmental conditions (Bowling and Bardo, 1994; Ewin et al., 2015), across rodent strains (Kosten et al., 1994; Watterson et al., 2015; Richardson et al., 2020), or across animals given various doses of a pharmacological agent (Hayes et al., 2009; Hachimine et al., 2014; Yates et al., 2021a). One simple analysis is to compare the time spent in the CS^+^ compartment during the posttest across experimental groups using an ANOVA (Poleszak and Malec, 2002). However, this approach fails to consider baseline differences that can exist across conditions; that is, one group may spend more time in the CS^+^ compartment during the pretest compared to another group. As such, this analysis is rarely used.

There are multiple ways to quantify CPP; however, these analytic approaches can be divided into two primary categories: (1) measuring the shift in time spent in the CS^+^ compartment from pretest to posttest and (2) comparing the time spent in the CS^+^ compartment to the time spent in the CS^–^ compartment during the pretest and the posttest. CPP is primarily quantified by applying a factorial ANOVA or a *t* test (or nonparametric equivalents) to determine if time spent in the CS^+^ compartment increases from pretest to posttest (Yates et al., 2021a) or to determine if the time spent in the CS^+^ compartment is higher than the time spent in the CS^–^ compartment during the posttest (Stojakovic et al., 2021). Imagine a researcher wants to determine if a pharmacological treatment differentially alters cocaine CPP in male and female rats living either in isolation or in social groups. If the researcher wants to examine two strains of rats, the analysis would be a four-way mixed factor ANOVA, with test period (or compartment if comparing the CS^+^ and CS^–^ compartments at posttest) as a within-subjects factor and sex, housing condition, and strain as between-subjects factors. To reduce the number of factors included in the analysis, one can calculate a difference score, thus eliminating test period (if comparing pretest to posttest) or compartment (if comparing the CS^+^ and CS^–^ compartments) as a factor. These difference scores often use the raw number of seconds spent in each compartment, but difference scores can be calculated using the percentage of time spent in each compartment (Carmack et al., 2013).

Somewhat related to difference scores is preference ratios. A preference ratio can be calculated by dividing the time spent in the CS^+^ compartment by the time spent in the CS^+^ and the CS^–^ compartments (Yates et al., 2013) or by dividing the CS^+^/CS^–^ difference score by the total time spent in each compartment (Weitz et al., 2021). Preference ratios can be expressed as the percentage of time in the CS^+^ compartment by multiplying the ratio by 100 (Luo et al., 2021). In cases in which multiple groups of subjects are tested, difference scores/preference ratios are analyzed with one-way or two-way ANOVAs (e.g., if a pharmacological intervention decreases preference for cocaine).

When using a two-compartment apparatus, calculating CPP is straightforward. If time in the CS^+^ compartment increases from pretest to posttest, the time spent in the CS^–^ must decrease across test period. When using an unbiased design, one consideration needs to be taken. If a subject spends more time in the CS^+^ compartment during the pretest compared to the CS^–^ compartment, calculating CPP by comparing the time spent in the CS^+^ to the CS^–^ during the posttest may not be appropriate. Suppose an animal spends 550 s in the CS^+^ compartment during a 900-s pretest and 600 s in the same compartment during the posttest. If the time in the CS^+^ compartment is compared to the time spent in the CS^–^ compartment during the posttest, the CPP score would be 300 s. However, the time spent in the CS^+^ increases by just 50 s from pretest to posttest. In this scenario, the researcher would want to either calculate a pre/post difference score or compare the time spent in the CS^+^ and the CS^–^ compartments during both the pretest and the posttest.

**FIGURE 2:**
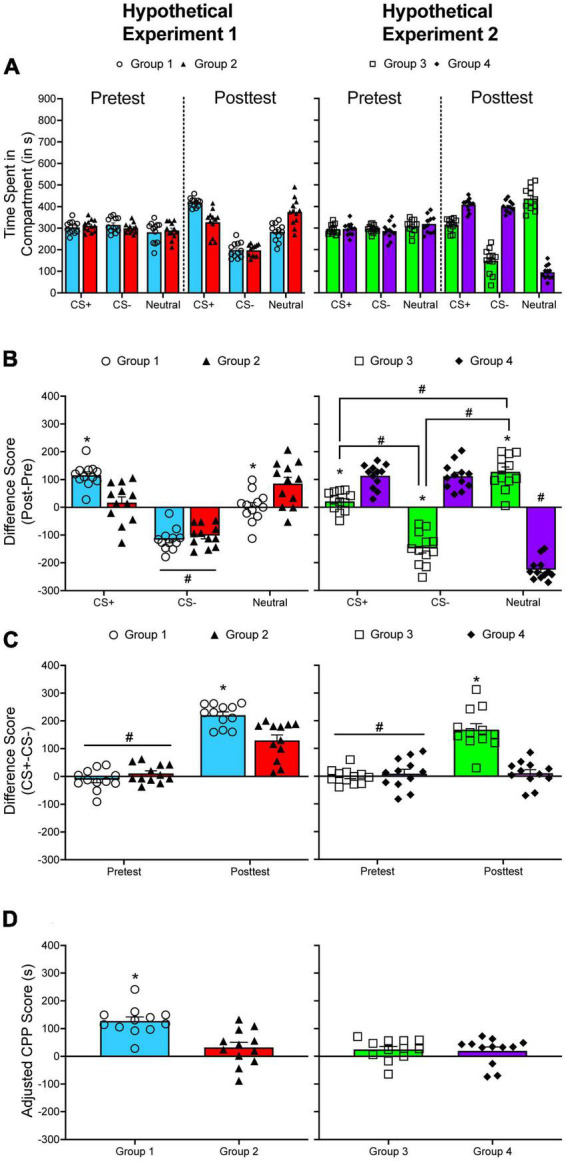
**(A)** The mean (±SEM) number of seconds spent in each compartment of a three-compartment CPP apparatus for two hypothetical experiments. **(B)** The mean (±SEM) difference in time spent for each compartment across the pretest and the posttest. *p < 0.05, compared to Group 2 (left panel) or compared to Group 4 (right panel). The # symbol indicates significant differences between compartments within the same group of subjects. **(C)** The mean (±SEM) difference in time spent in the CS^+^ and the CS^–^ compartments during the pretest and during the posttest. *p < 0.05, compared to Group 2 (left panel) or compared to Group 4 (right panel). #*p* < 0.05, compared to the posttest. **(D)** Mean (±SEM) adjusted CPP scores. *p < 0.05, compared to Group 2 (left panel).

Calculating CPP becomes more complicated when using a three-compartment apparatus. Figure 2 shows two hypothetical datasets generated from an unbiased three-compartment apparatus. Data for two groups of subjects were generated using *R* for each dataset (*n* = 12 per group per dataset). The raw data presented in this figure are normally distributed (Figure 1A). During the pretest, the time spent in each compartment is nearly equivalent for each group. For the first hypothetical dataset, the time spent in the CS^+^ compartment increases while time spent in the CS^–^ compartment decreases for Group 1 from pretest to posttest. The time spent in the neutral compartment does not change. Like Group 1, the time spent in the CS^–^ compartment decreases for Group 2; however, the time spent in the CS^+^ compartment does not change significantly from pretest to posttest. Instead, the time spent in the neutral compartment increases. If CPP is quantified by comparing either the shift in time spent in the CS^+^ across test period or the time spent in the CS^+^ and the CS^–^ during the posttest, the interpretation is identical: Group 1 develops greater CPP compared to Group 2 (Figures 2B, C, left panel).

In the hypothetical situation presented here, CS^+^_*post*_/CS^+^_*pre*_ difference scores and CS^+^_*post*_/CS^–^_*post*_ difference scores are positively and significantly correlated, *r*(22) = 0.867, *p* < 0.001. Table 1 presents the correlation between CS^+^_*post*_/CS^+^_*pre*_ difference scores and CS^+^_*post*_/CS^–^_*post*_ difference scores using data I have collected from different CPP experiments. Overall, the correlation between each analysis is positive and statistically significant, mirroring the results of the hypothetical dataset.

**TABLE 1:** Correlations between CS^+^_post_/CS^–^_post_ and CS^+^_post_/CS^+^_pre_ difference scores for several CPP experiments conducted by the author of this paper.

CS^+^	Type of design	Correlation between CS^+^-CS^–^ and post-pre analysis	*p*-value	References
AMPH	Biased	*r*(34) = 0.843	<0.001	Yates et al., 2012
AMPH	Unbiased	*r*(22) = 0.649	<0.001	Yates et al., 2013
Social interaction	Unbiased	*r*(22) = 0.678	<0.001	Yates et al., 2013
AMPH vs. social interaction	Unbiased	*r*(22) = 0.671	<0.001	Yates et al., 2013
METH	Biased	*r*(82) = 0.840	<0.001	Yates et al., 2021a
Cocaine	Biased	*r*(89) = 0.884	<0.001	Yates et al., 2021b
AMPH	Biased	*r*(6) = 0.954	<0.001	Unpublished results
METH	Biased	*r*(30) = 0.681	<0.001	Unpublished results
METH	Biased	*r*(125) = 0.801	<0.001	Unpublished results

AMPH, amphetamine; METH, methamphetamine.

While there is often high concordance between analyses, there are situations in which analytic approaches can lead to discrepant conclusions. In the second hypothetical dataset (right column of Figure 2), if the difference in time spent in the CS^+^ compartment from pretest to posttest is compared between each group (Figure 2B), the results indicate that Group 4 develops greater CPP compared to Group 3. However, if the difference in time spent in the CS^+^ and the CS^–^ compartment is used, the results indicate that Group 3 shows greater CPP than Group 4 (Figure 2C).

One potential issue surrounding the use of different analyses to quantify CPP is replicability. In the 1990s, two studies examined the effects of naltrindole, a delta opioid receptor antagonist, on cocaine CPP (Menkens et al., 1992; de Vries et al., 1995). Both studies used male rats (Lewis rats in the former study and Sprague Dawley rats in the latter study) as subjects and used an apparatus with two compartments. de Vries et al. (1995) used a lower dose of cocaine (10.0 mg/kg) compared to Menkens et al. (1992) (15.0 mg/kg), but at least one group of rats in each experiment received 3.0 mg/kg of naltrindole. Whereas Menkens et al. (1992) found that naltrindole decreased cocaine CPP, de Vries et al. (1995) did not replicate this finding. Determining if the discrepancy observed across studies resulted from strain differences and/or different cocaine doses is difficult as the way CPP was quantified varied across studies. de Vries et al. (1995) compared the total time spent in the cocaine-paired compartment across experimental groups. Menkens et al. (1992) calculated the percentage change in time spent in the cocaine-paired compartment from pretest to posttest before comparing experimental groups. Because neither study presented the time spent in each compartment during the pretest and during the posttest, this further increases the difficulty of directly comparing the results of these studies.

Discrepant results have also been observed when examining sex differences in CPP. Using Sprague Dawley rats, Cicero et al. (2000) did not observe strong sex differences in morphine CPP at doses of 0.2–7.5 mg/kg. Between 10.0 and 17.5 mg/kg, females showed greater CPP. However, Karami and Zarrindast (2008) observed greater CPP in Wistar females relative to males at low doses (0.5–7.5 mg/kg), an effect that disappeared at 10.0 mg/kg. Determining if the discrepancy across studies reflects a strain difference, a difference in apparatus type (three vs. two compartments), and/or a procedural difference (biased vs. unbiased design) is difficult. This difficulty is compounded by differential analytic approaches observed across studies. Cicero et al. (2000) quantified CPP with the equation:


(1) (CS-+p⁢o⁢s⁢tCS)-p⁢o⁢s⁢t-(CS-+p⁢r⁢eCS)-p⁢r⁢e

while Karami and Zarrindast calculated the difference in time spent in the CS^+^ compartment only. Once again, directly comparing these results is difficult because the time spent in each compartment is not presented in either paper presenting these results.

Recently, discrepancies have been reported concerning sex differences in oxycodone CPP from the same group of authors. When CPP was quantified as a percentage of time spent in the oxycodone-paired compartment during the posttest, female rats developed greater CPP compared to males (Ryan et al., 2018). Yet, when CPP was quantified as a percentage change in time spent in the oxycodone-paired compartment from pretest to posttest, no significant sex differences were observed (Randesi et al., 2019), although interestingly, males had a slightly larger percentage change score relative to females. Because these studies were conducted by the same group of researchers, all other aspects of the experimental procedures were consistent (e.g., use of Sprague Dawley rats, use of a biased design, same dose of oxycodone). This example provides more concrete evidence as to how the analytic approach can alter one’s interpretation of CPP.

## Materials and equipment

There are no special materials or equipment needed to perform the analyses described in this paper. I have included an Excel file that provides a template for calculating adjusted CPP scores (see Supplementary material). This Excel file also contains the raw data used in the datasets described below.

### Methods

I recommend using an analysis that considers the change in time spent in the CS^+^ compartment from pretest to posttest and the difference in time spent in the CS^+^ and the CS^–^ compartment during the posttest. Numerous studies already compare the time spent in the CS^+^ compartment to the time spent in the CS^–^ compartment at pretest and at posttest (see Supplementary Table 1). Some experiments use Equation 1 from above, which incorporates each difference score (Cicero et al., 2000; Cooper et al., 2021; Avelar et al., 2022). When this equation is used to quantify CPP for the hypothetical data presented in Figure 2, there is a significant difference in CPP scores between each group in the first hypothetical example, with Group 1 (232.017 ± 16.831 s) showing increased CPP compared to Group 2 (118.825 ± 24.275 s). In the second hypothetical dataset, Group 3 (169.013 ± 22.217 s) shows increased CPP compared to Group 4 (1.384 ± 23.674 s), which is consistent with the CS^+^_*post*_/CS^–^_*post*_ analysis presented above.

One unique challenge associated with drug CPP is dissociating true preference for the drug-paired compartment from increased novelty seeking (Bardo and Bevins, 2000). An animal that has been subjected to repeated pairings of a drug like cocaine and a specific environmental context may not recall being in the CS^+^ compartment at any point before the posttest. Therefore, the animal may treat the CS^+^ compartment like a novel environment (Tzschentke, 1998). The three-compartment apparatus is used to control for the influence of novelty seeking on CPP (Bardo and Bevins, 2000); yet, current analytic approaches consistently fail to include how the time spent in the neutral compartment changes from pretest to posttest. Below I present the time spent in the CS^+^ and the CS^–^ compartments for one subject that was tested for social interaction CPP using an unbiased design (Yates et al., 2013):


CS=+pre333.14,CS=-pre263.15,CSpost+

=341.82,andCS=-post197.95

If a difference score is calculated for the posttest only, the CPP score is 143.87 s. If Equation 1 is used, the CPP score is reduced to 73.88 s. However, if just the change in time spent in the CS^+^ compartment from pretest to posttest is calculated, the CPP score decreases significantly to 8.68 s. Even though the example data presented for the individual subject above comes from a social CPP experiment, the influence of novelty seeking can be observed. The time spent in the neutral compartment increases from 303.71 to 360.23 s for this subject.

To address the potential limitation described above, a different equation can be used to quantify CPP:


(2) $$\Psi =\frac{\rho +\Lambda }{2},\mathrm{where}$$

$$\rho =\left(\left({\text{CS}}_{\mathrm{post}}^{+}-{\text{CS}}_{\mathrm{post}}^{-}\right)-\left({\text{CS}}_{\mathrm{pre}}^{+}-{\text{CS}}_{\mathrm{pre}}^{-}\right)\right)$$

$$-\left(Y\times \left(1-\left(\frac{\text{Y}}{\text{Y}+X+{\text{Z}}_{\alpha }+{\text{Z}}_{\beta }}\right)\right)\right)$$

$$\text{and}\Lambda =\left({\text{CS}}_{\mathrm{post}}^{+}-{\text{CS}}_{\mathrm{pre}}^{+}\right)-(\left(\frac{\left({\text{CS}}_{\mathrm{post}}^{+}-{\text{CS}}_{\mathrm{pre}}^{+}\right)+\left|{\text{CS}}_{\mathrm{post}}^{+}-{\text{CS}}_{\mathrm{pre}}^{+}\right|}{2}\right)\times$$

$$\left(1-\left(\frac{\frac{\frac{\left({\text{CS}}_{\mathrm{post}}^{+}-{\text{CS}}_{\mathrm{pre}}^{+}\right)+\left|{\text{CS}}_{\mathrm{post}}^{+}-{\text{CS}}_{\mathrm{pre}}^{+}\right|}{2}}{{\text{CS}}_{\mathrm{pre}}^{+}}}{\begin{array}{c} \frac{\frac{\left({\text{CS}}_{\mathrm{post}}^{+}-{\text{CS}}_{\mathrm{pre}}^{+}\right)+\left|{\text{CS}}_{\mathrm{post}}^{+}-{\text{CS}}_{\mathrm{pre}}^{+}\right|}{2}}{{\text{CS}}_{\mathrm{pre}}^{+}}+\frac{\frac{\left({\text{CS}}_{\mathrm{post}}^{-}-{\text{CS}}_{\mathrm{pre}}^{-}\right)+\left|{\text{CS}}_{\mathrm{post}}^{-}-{\text{CS}}_{\mathrm{pre}}^{-}\right|}{2}}{{\text{CS}}_{\mathrm{pre}}^{-}}\\ +\frac{\frac{\left({\text{N}}_{\mathrm{post}}-{\text{N}}_{\mathrm{pre}}\right)+\left|{\text{N}}_{\mathrm{post}}-{\text{N}}_{\mathrm{pre}}\right|}{2}}{{\text{N}}_{\mathrm{pre}}} \end{array}}\right)\right))$$

To calculate ρ, Equation 1 is modified to include a penalizer term. The letters Y, X, Z_α_, and Z_β_ represent separate difference scores:


$$\text{Y}=\frac{\begin{array}{c} \left(\left({\text{CS}}_{\mathrm{post}}^{+}-{\text{CS}}_{\mathrm{post}}^{-}\right)-\left({\text{CS}}_{\mathrm{pre}}^{+}-{\text{CS}}_{\mathrm{pre}}^{-}\right)\right)\\ +\left|\left({\text{CS}}_{\mathrm{post}}^{+}-{\text{CS}}_{\mathrm{post}}^{-}\right)-\left({\text{CS}}_{\mathrm{pre}}^{+}-{\text{CS}}_{\mathrm{pre}}^{-}\right)\right| \end{array}}{2},$$

$$\text{X}=\frac{\begin{array}{c} \left(\left({\text{N}}_{\mathrm{post}}-{\text{CS}}_{\mathrm{post}}^{+}\right)-\left({\text{N}}_{\mathrm{pre}}-{\text{CS}}_{\mathrm{pre}}^{+}\right)\right)\\ +\left|\left({\text{N}}_{\mathrm{post}}-{\text{CS}}_{\mathrm{post}}^{+}\right)-\left({\text{N}}_{\mathrm{pre}}-{\text{CS}}_{\mathrm{pre}}^{+}\right)\right| \end{array}}{2},$$

$${\text{Z}}_{\alpha }=\frac{\begin{array}{c} \left(\left({\text{N}}_{\mathrm{post}}-{\text{CS}}_{\mathrm{post}}^{-}\right)-\left({\text{N}}_{\mathrm{pre}}-{\text{CS}}_{\mathrm{pre}}^{-}\right)\right)\\ +|\left(\left({\text{N}}_{\mathrm{post}}-{\text{CS}}_{\mathrm{post}}^{-}\right)-\left({\text{N}}_{\mathrm{pre}}-{\text{CS}}_{\mathrm{pre}}^{-}\right)\right| \end{array}}{2},\text{and}$$

$${\text{Z}}_{\beta }=\frac{\begin{array}{c} \left(\left({\text{CS}}_{\mathrm{post}}^{-}-{\text{N}}_{\mathrm{post}}\right)-\left({\text{CS}}_{\mathrm{pre}}^{-}-{\text{N}}_{\mathrm{pre}}\right)\right)\\ +\left|\left({\text{CS}}_{\mathrm{post}}^{-}-{\text{N}}_{\mathrm{post}}\right)-\left({\text{CS}}_{\mathrm{pre}}^{-}-{\text{N}}_{\mathrm{pre}}\right)\right| \end{array}}{2}.$$

If the CS^+^ is rewarding, the time spent in the CS^+^ compartment should increase from pretest to posttest, and the time spent in the CS^–^ compartment should decrease across each test session. The time in the neutral (represented by N above) compartment should ideally remain unchanged or should decrease. If subjects show a greater difference in time spent in the neutral compartment relative to either the CS^+^ compartment or the CS^–^ compartment from pretest to posttest, the penalizer will increase. Thus, one purpose of this equation is to penalize subjects that show increased novelty seeking-like behavior. Notice that Z_α_ and Z_β_ are the opposite of one another, meaning that at least one of these equations will equal 0. However, Z_β_ is included to determine if subjects show concomitant increases in time spent in the CS^+^ and the CS^–^ compartments. In the event that a subject spends less time in the CS^+^ compartment relative to the CS^–^ compartment, ρ reduces to Equation 1.

The calculation for Λ is somewhat similar to the one used to derive ρ. This equation focuses on the change in time spent in each individual compartment from pretest to posttest. Like the equation for ρ, the difference in time spent in the CS^+^ compartment from pretest to posttest is subtracted by a penalizer term. The first part of the penalizer term adds the CS^+^ pre/post difference score to the absolute value of itself before being divided by 2. This ensures that a negative value is set to 0, thus making ρ equal to CS^+^_*post*_ – CS^+^_*pre*_. In this event, there is no need to penalize an animal that spends less time in the CS^+^ compartment at posttest relative to pretest. If the time spent in the CS^+^ compartment increases from pretest to posttest, then the increase in time spent in this compartment is divided by the increase in time spent in the CS^–^ compartment or the neutral compartment. Like Z_α_ and Z_β_, at least one expression will equal 0 as a subject cannot increase their time spent in all three compartments from pretest to posttest. In a situation in which the time spent in the CS^+^ compartment increases only, Λ reduces to CS^+^_*post*_ – CS^+^_*pre*_. As the difference in time spent in the CS^–^ compartment or the neutral compartment from pretest to posttest increases in magnitude relative to the difference in time spent in the CS^+^ compartment, the penalizer term increases, thus decreasing Λ.

Figure 2D shows adjusted CPP scores for each hypothetical experiment described above. Not surprisingly, adjusted CPP scores are higher for Group 1 (135.704 s) compared to Group 2 (31.552 s) in the first hypothetical experiment. However, adjusted CPP scores do not differ across each group for the second experiment (28.154 s vs. 18.259 s).

### Applying the adjusted CPP score to previously published data

I have chosen to reanalyze three experiments that quantified CPP in different ways. For each data set, I apply several analytic approaches: (1) ANOVA comparing the difference in time spent from pretest to posttest for both CS^+^ and CS^–^ compartments; (2) ANOVA comparing the difference in time spent in the CS^+^ and the CS^–^ compartments for both pretest and posttest, and (3) ANOVA comparing adjusted CPP scores.

In the first study (Yates et al., 2012), rats were tested in a behavioral measure of impulsivity before being tested for amphetamine CPP using a biased design. Three different experiments were conducted as we tested three doses of amphetamine: 0.1, 0.5, and 1.5 mg/kg. In the second study (Yates et al., 2013), we conducted three experiments to compare the conditioned rewarding effects of social interaction and amphetamine across differentially housed adolescent and adult rats. In one experiment, we examined social interaction CPP. In another experiment, we measured amphetamine CPP. The third experiment will be discussed in a different section. For each experiment, we used an unbiased design. In the third study (Yates et al., 2021a), we aimed to determine if the drug Ro 63-1908 blocks the acquisition and/or the expression of methamphetamine CPP. To test the effects of Ro 63-1908 on the acquisition of methamphetamine CPP, separate groups of rats received Ro 63-1908 (0, 1.0, 3.0 mg/kg; note, an additional group of females received 10.0 mg/kg) before each methamphetamine conditioning session. To test the effects of Ro 63-1908 on the expression of CPP, rats received Ro 63-1908 (0, 1.0, 3.0, 10.0 mg/kg) prior to the posttest.

## Results

**FIGURE 3:**
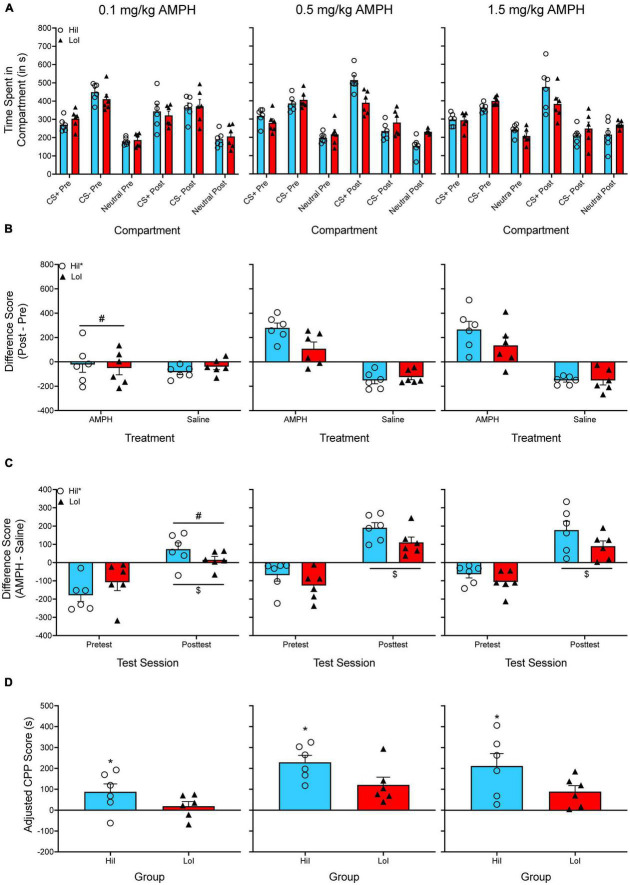
**(A)** The mean (±SEM) number of seconds spent in each compartment of a three-compartment CPP apparatus for three groups of rats treated with one dose of amphetamine (AMPH; 0.1, 0.5, or 1.5 mg/kg). AMPH was paired with the CS^+^ compartment while saline was paired with the CS^–^ compartment. Animals were previously screened for impulsive choice and were classified as either high impulsive (HiI) or low impulsive (LoI). **(B)** The mean (±SEM) difference in time spent for the AMPH-paired and the saline-paired compartments across the pretest and the posttest. *p < 0.05, indicates a main effect of impulsivity. #*p* < 0.05, indicates that animals treated with the lowest dose of AMPH (0.1 mg/kg) spent less time in the AMPH-compartment compared to animals treated with the higher doses of AMPH. **(C)** The mean (±SEM) difference in time spent in the AMPH-paired and the saline-paired compartments during the pretest and during the posttest. *p < 0.05, indicates a main effect of impulsivity. #*p* < 0.05, indicates that animals treated with the lowest dose of AMPH (0.1 mg/kg) had lower difference scores during the posttest compared to animals treated with the higher doses of AMPH. $*p* < 0.05, compared to the pretest. **(D)** Mean (±SEM) adjusted CPP scores. *p < 0.05, indicates a main effect of impulsivity.

Yates et al. (2012). For each experiment, we originally calculated a difference score by subtracting the time spent in the amphetamine-paired compartment by the time spent in the saline-paired compartment during the posttest. Difference scores were analyzed with a two-way ANOVA, with amphetamine dose and impulsivity as between-subjects factors. Figure 3A shows the time spent in each compartment for both the pretest and the posttest.

Figure 3B shows the difference in time spent in the CS^+^ compartment from pretest to posttest. The difference in time spent in the CS^–^ compartment from pretest to posttest is also plotted but is not included in the analysis, which is common for experiments using CS^+^_*post*_/CS^+^_*pre*_ difference scores. A two-way ANOVA is used to analyze CS^+^_*post*_/CS^+^_*pre*_ difference scores, with amphetamine dose and impulsivity as between-subjects factors. There are two main effects, *F*(2, 30) = 10.214, *p* < 0.001 and *F*(1, 30) = 5.170, *p* = 0.030, but no significant interaction.

Figure 3C shows difference scores as originally published in Yates et al. (2012). Unlike the Yates et al. (2012) paper, I have additionally included difference scores for the pretest. A mixed factor ANOVA is used to analyze these data, with test period as the within-subjects factor and amphetamine dose and impulsivity as between-subjects factors. Once again, there are main effects of amphetamine dose and impulsivity, *F*(2, 30) = 7.879, *p* = 0.002 and *F*(1, 30) = 5.754, *p* = 0.023, as well as a main effect of test period, *F*(1, 30) = 115.058, *p* < 0.001. However, there are no significant interactions.

Figure 3D shows adjusted CPP scores. These data are analyzed with a two-way ANOVA, with amphetamine dose and impulsivity as between-subjects factors. There are main effects of dose, *F*(2, 30) = 8.889, *p* < 0.001, and impulsivity, *F*(1, 30) = 7.964, *p* = 0.008, but no significant interaction.

Yates et al. (2013). We originally quantified CPP with a preference ratio, calculated as $\frac{{\text{CS}}^{+}}{{\text{CS}}^{+}+{\text{CS}}^{-}}$. For simplicity, I will present the data from the amphetamine CPP experiment.

**FIGURE 4:**
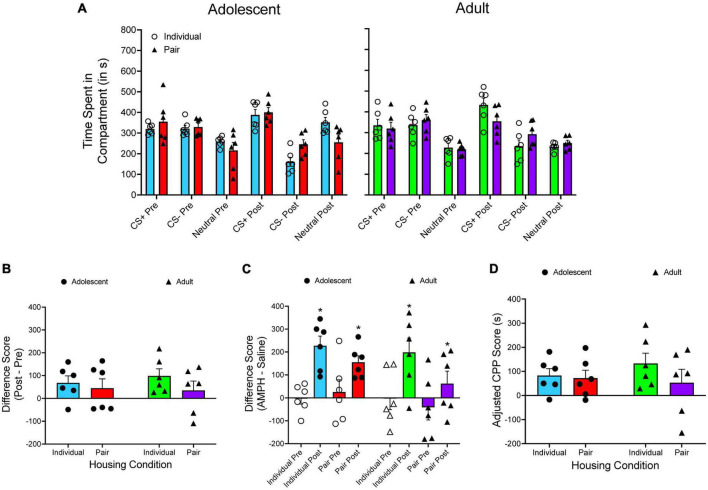
**(A)** The mean (±SEM) number of seconds spent in each compartment of a three-compartment CPP apparatus for adolescent rats (left panel) and adult rats (right panel) that were either individually housed (denoted by open circles) or pair-housed (denoted by closed triangles). Amphetamine (AMPH) was paired with the CS^+^ compartment while saline was paired with the CS^–^ compartment. **(B)** The mean (±SEM) difference in time spent for the AMPH-paired and the saline-paired compartments across the pretest and the posttest. **(C)** The mean (±SEM) difference in time spent in the AMPH-paired and the saline-paired compartments during the pretest and the posttest. *p < 0.05, compared to the pretest. **(D)** Mean (±SEM) adjusted CPP scores.

The time spent in each compartment is presented in Figure 4A. A two-way ANOVA is used to analyze CS^+^_*post*_/CS^+^_*pre*_ difference scores (Figure 4B), with housing condition and age as between-subjects factors. There are no main effects of housing condition or age, as well as no significant interaction. When the time spent in the CS^–^ compartment is subtracted from the time spent in the CS^+^ compartment (Figure 4C), a mixed factor ANOVA reveals a main effect of test period only, *F*(1, 20) = 25.632, *p* < 0.001. When adjusted CPP scores are analyzed (Figure 4D), there are no main effects and no significant interaction.

**FIGURE 5:**
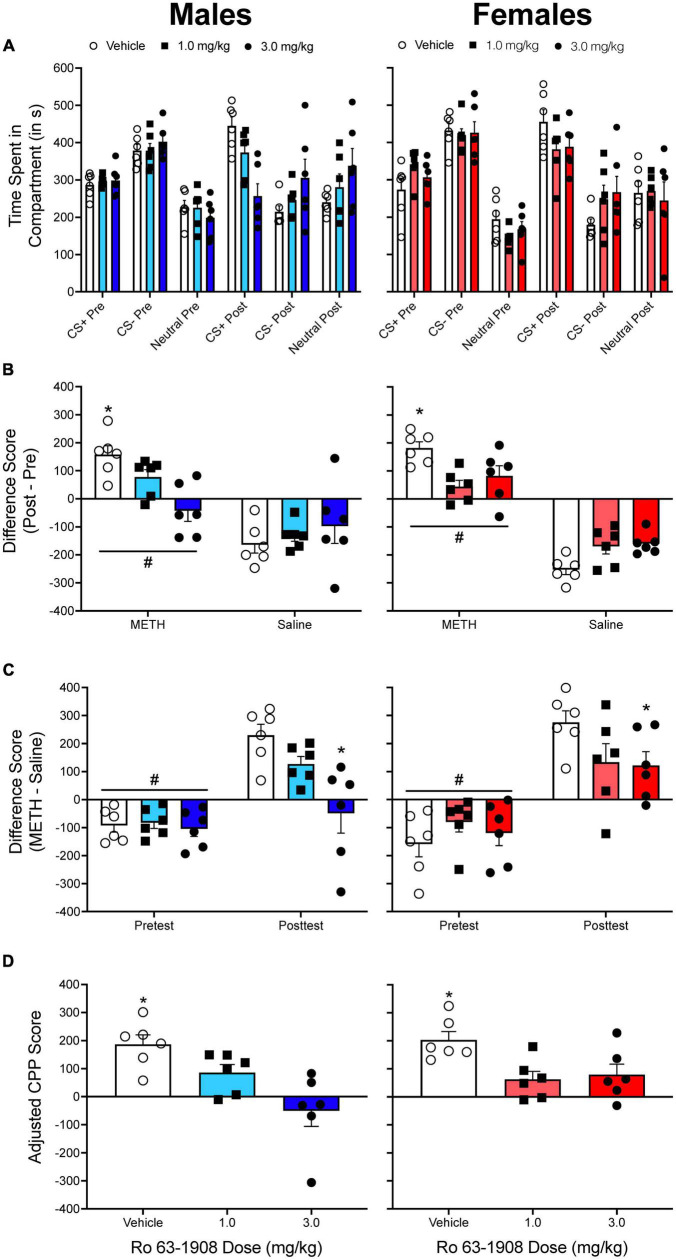
**(A)** The mean (±SEM) number of seconds spent in each compartment of a three-compartment CPP apparatus for males (left column) and for females (right column) pretreated with one of three doses of the drug Ro 63-1908 (vehicle, 1.0, and 3.0 mg/kg). Methamphetamine (METH) was paired with the CS^+^ compartment while saline was paired with the CS^–^ compartment. **(B)** The mean (±SEM) difference in time spent in the METH-paired and the saline-paired compartments across the pretest and the posttest. *p < 0.05, compared to rats pretreated with each dose of Ro 63-1908. #*p* < 0.05, compared to the saline-paired compartment. **(C)** The mean (±SEM) difference in time spent in the METH-paired and the saline-paired compartments during the pretest and the posttest. *p < 0.05, compared to vehicle-pretreated rats. #*p* < 0.05, compared to the posttest. **(D)** Mean (±SEM) adjusted CPP scores. *p < 0.05, compared to rats pretreated with each dose of Ro 63-1908.

Yates et al. (2021a). We originally used a mixed factor ANOVA to analyze the time spent in the methamphetamine-paired compartment during the pretest and during the posttest. For simplicity, I have reanalyzed the data for the acquisition experiment, as we found significant effects of Ro 63-1908 on the acquisition of methamphetamine CPP, an effect that was more pronounced in male rats. The time spent in each compartment is presented in Figure 5A.

When pretest/posttest difference scores are analyzed (Figure 5B), there is a main effect of compartment, *F*(1, 30) = 138.775, *p* < 0.001, and significant compartment × sex, *F*(1, 30) = 5.881, *p* = 0.022, and compartment × Ro 63-1908 dose, *F*(2, 30) = 10.787, *p* < 0.001, interactions. When CS^+^/CS^–^ difference scores are analyzed (Figure 5C), there are main effects of test period, *F*(1, 30) = 138.775, *p* < 0.001, and Ro 63-1908 dose, *F*(2, 30) = 4.218, *p* < 0.024. There are also significant interactions between test period and sex, *F*(1, 30) = 5.881, *p* = 0.022, and test period and Ro 63-1908 dose, *F*(2, 30) = 10.787, *p* < 0.001. Figure 5D shows adjusted CPP scores. A two-way ANOVA reveals a main effect of Ro 63-1908 dose, *F*(2, 30) = 12.363, *p* < 0.001. When the data are collapsed across sex, each dose of Ro 63-1908 decreases adjusted CPP scores.

### Summary of results

Overall, there was consistency in the results obtained from each analysis performed for the three experiments described above. High impulsive animals develop greater amphetamine CPP compared to low impulsive animals (Yates et al., 2012), amphetamine CPP does not significantly differ between individually and pair-housed adolescent or adult rats (Yates et al., 2013), and the drug Ro 63-1908 decreases methamphetamine CPP in both males and females (Yates et al., 2021a). There was one minor exception that was observed in the Yates et al. (2021a) study. When CS^+^/CS^–^ difference scores were analyzed, the results showed that only the high dose of Ro 63-1908 (3.0 mg/kg) decreased methamphetamine CPP. When pre/post difference scores or adjusted CPP scores were analyzed, results indicated that both doses of Ro 63-1908 decreased methamphetamine CPP.

There was one interesting difference that emerged when applying different analytic approaches to previously published data. When examining amphetamine CPP in adolescent and adult rats (Yates et al., 2013), the results of the CS^+^/CS^–^ difference score suggest that each group has a larger difference score at posttest relative to pretest, thus indicating CPP has occurred. However, when inspecting pre/post difference scores and adjusted CPP scores, the magnitude of CPP is greatly blunted. Specifically, no group had a mean pre/post difference score higher than 100 s. When adjusted CPP scores are examined, only individually housed adult rats had a score above 100 s (132.953 s). Even though an unbiased design was used in this experiment, the current results emphasize one of the issues of using CS^+^/CS^–^ difference scores to quantify CPP. Looking at Figure 4A, one can see that the time spent in the neutral compartment increases from pretest to posttest for both adolescent groups, particularly for individually housed adolescents.

When examining raw data (i.e., time spent in each compartment), the percentage increase in time spent in the neutral compartment from pretest to posttest is greater than the percentage increase in time spent in the CS^+^ for each group (21.391% vs. 35.881%, 12.804% vs. 17.941%, and 13.714% vs. 11.0442%), with the exception of individually housed adult rats (29.316% vs. 1.588%). These results appear to suggest that adolescent rats are particularly sensitive to novelty seeking, thus further highlighting the need to control for changes in time spent in the neutral compartment when quantifying CPP using a three-compartment apparatus.

### Use of adjusted CPP scores when no true CS^–^ is included

Some CPP experiments focus on comparing choice for one stimulus relative to another stimulus (e.g., drug vs. social interaction) (Yates et al., 2013; Zernig et al., 2013; Kummer et al., 2014). In the third experiment conducted by Yates et al. (2013), rats were tested for concurrent choice between social interaction and amphetamine. In a concurrent-choice CPP experiment, there is no true CS^–^. Each compartment is paired with a stimulus that can elicit a conditioned approach response. In the Yates et al. (2013) experiment, the preference ratio included the time spent in the social-paired compartment in the numerator of the preference ratio. If the amphetamine-paired compartment was used in the numerator, the values of the preference ratio would have been $1-\frac{\text{Social}}{\text{Social}+\mathrm{Amphetamine}}$.

**FIGURE 6:**
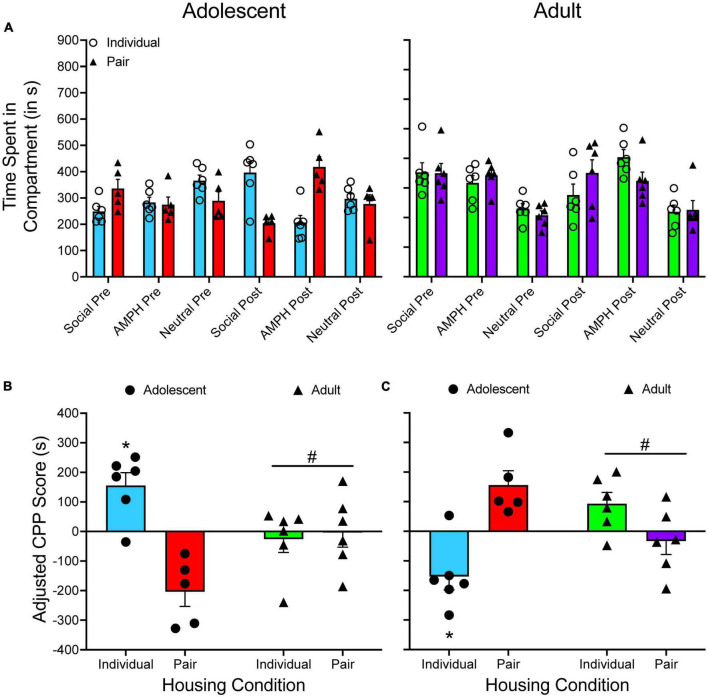
**(A)** The mean (±SEM) number of seconds spent in each compartment of a three-compartment CPP apparatus for adolescent rats (left panel) and adult rats (right panel) that were either individually housed (denoted by open circles) or pair-housed (denoted by closed triangles). In this experiment, there was no true CS^–^ as rats learned to associate one compartment with amphetamine (AMPH) and one compartment with social interaction. **(B)** Mean (±SEM) adjusted CPP scores when social interaction is treated as the CS^+^. **(C)** Mean (±SEM) adjusted CPP scores when AMPH is treated as the CS^+^. *p < 0.05, compared to pair-housed rats. #*p* < 0.05, relative to housing-matched adolescent rats.

Figure 6A shows the time spent in each compartment for both adolescent and adult subjects that were either individually housed or pair housed. Figure 6B shows adjusted CPP scores when social interaction is treated as the CS^+^ while Figure 6C shows adjusted CPP scores when amphetamine is treated as the CS^+^. Due to the way the adjusted CPP score is calculated, primarily how it treats negative values, reversing which compartment is considered the CS^+^ compartment leads to slight alterations to the adjusted CPP score. For example, when examining individually housed adult rats, the adjusted CPP score is −25.764 s when social interaction is entered as the CS^+^ compartment. When social interaction is treated as the CS^–^, the adjusted CPP becomes 93.351 s.

Despite the discrepancy in adjusted CPP scores, the results of each two-way ANOVA are similar, regardless of which stimulus is classified as the CS^+^. There is one subtle difference in the results of the analysis. When social interaction is the CS^+^, there is a main effect of housing, *F*(1, 19) = 12.543, *p* = 0.002, and a significant age × housing interaction, *F*(1, 19) = 16.380, *p* < 0.001. When amphetamine is the CS^+^, there is a significant interaction, *F*(1, 19) = 24.382, *p* < 0.001, but the main effect of housing approaches significance only, *F*(1, 19) = 4.225, *p* = 0.053. However, probing the significant interaction leads to the same conclusions: pair-housed adolescents spend significantly more time in the compartment paired with amphetamine compared to individually housed adolescents, with adults spending similar amounts of time in both compartments.

### Quantifying extinction and reinstatement with the adjusted CPP score

In addition to measuring the conditioned rewarding effects of a stimulus, CPP can be used to study relapse-like behavior. Following the posttest, subjects are given extinction training. There are two ways in which extinction training can occur. First, subjects are allowed to explore each compartment of the CPP apparatus as in the pretest/posttest. This method allows one to measure the rate at which conditioned approach to the CS^+^ compartment returns to pretest levels. In the second method, subjects are isolated to the CS^+^ and CS^–^ compartments on alternating sessions as during the conditioning phase of the experiment. Before being placed in each compartment, the animal receives an injection of vehicle. After a certain number of sessions, subjects are given a test session to determine if the time spent in the CS^+^ compartment has returned to pretest levels. If not, subjects receive additional extinction sessions before receiving another test session. Once extinction has occurred, subjects are given a reinstatement test. In drug-induced reinstatement, subjects are given a priming injection of the drug that served as the CS^+^ before being placed in the CPP apparatus. In stress-induced reinstatement, subjects are exposed to a stressor like restraint before being placed in the CPP apparatus.

**FIGURE 7:**
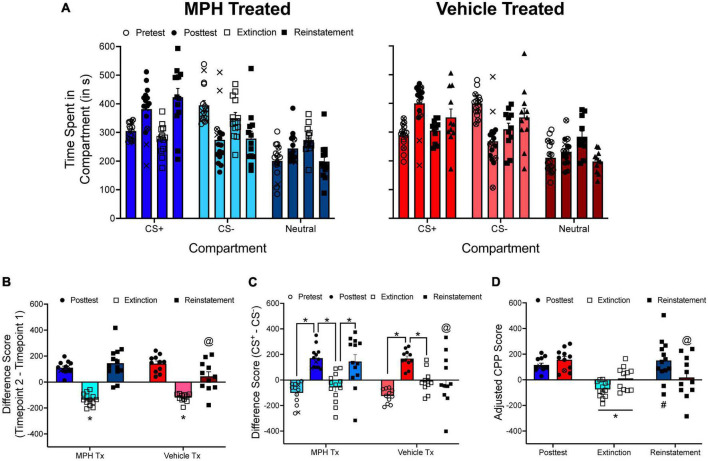
**(A)** The mean (±SEM) number of seconds spent in each compartment of a three-compartment CPP apparatus for rats treated with either methylphenidate (MPH; left panel) or vehicle (right panel) during adolescence. Rats were tested for methamphetamine (METH) CPP during adulthood. Following the posttest, rats were given extinction training followed by a reinstatement test. **(B)** The mean (±SEM) difference in time spent for the METH-paired compartment from pretest to posttest, from posttest to the end of extinction training, and from the end of extinction training to the reinstatement test. *p < 0.05, compared to difference scores calculated at the end of the posttest and following the reinstatement test. @*p* < 0.05, compared to MPH-treated rats. **(C)** The mean (±SEM) difference in time spent in the METH-paired and the saline-paired compartments across each phase of the experiment. The * indicates that difference scores significantly differed from one phase of the experiment to the next phase of the experiment. @*p* < 0.05, compared to MPH-treated rats. **(D)** Mean (±SEM) adjusted CPP scores calculated at the end of the posttest, at the end of extinction training, and following the reinstatement test. *p < 0.05, compared to the posttest. #*p* < 0.05, compared to the end of extinction. @*p* < 0.05, compared to MPH-treated rats. X’s indicate rats that spent less time in the CS^+^ compartment during the posttest compared to the pretest. These subjects were not tested for extinction or reinstatement of METH CPP. Circles with an X in them indicate subjects that failed to meet extinction criteria after 60 sessions.

Figure 7 shows unpublished data from a reinstatement experiment conducted in my laboratory. In this experiment, spontaneously hypertensive rats (SHRs), an animal model of attention-deficit/hyperactivity disorder (ADHD), and Wistar-Kyoto rats (WKYs), the inbred control strain to SHRs, received oral administration of either methylphenidate or vehicle during adolescence before being tested for methamphetamine CPP (1.0 or 2.0 mg/kg) during adulthood. We originally quantified CPP by examining the change in time spent in the methamphetamine-paired compartment from pretest to posttest. To extinguish CPP, we exposed rats to all three compartments of the CPP apparatus in a drug-free state until specific criteria were met.

For illustrative purposes, data for female SHRs will be presented only. The time spent in each compartment is presented in Figure 7A. The time spent in the CS^+^ compartment can be directly compared across phases of the CPP experiment (Figure 7B), or the difference in time spent in the CS^+^ and CS^–^ compartments can be directly compared across each phase of the CPP experiment (Figure 7C). When pre/post difference scores are analyzed, there is a main effect of test period, *F*(2, 44) = 58.746, *p* < 0.001, and a significant test period × adolescent treatment interaction, *F*(2, 44) = 4.027, *p* = 0.025. Likewise, when CS^+^/CS^–^ difference scores are analyzed, there is a main effect of test period, *F*(3, 66) = 28.010, *p* < 0.001, and a significant interaction between test period and adolescent treatment, *F*(3, 66) = 3.040, *p* = 0.035. Overall, difference scores increase after the posttest and the reinstatement test, and decrease following extinction training. However, there is some discrepancy with how data are interpreted if using pre/post difference scores or CS^+^/CS^–^ difference scores. When pre/post difference scores are used, the results indicate that both methylphenidate- and vehicle-treated rats showed reinstatement of methamphetamine CPP. Yet, when CS^+^/CS^–^ difference scores are used, methylphenidate-treated rats show reinstatement of CPP only.

Equation 2 can be modified to quantify extinction of CPP:


(3) $$\Psi_{e}=\Psi +\frac{\rho_{\text{e}}+\Lambda_{\text{e}}}{2}$$

One major addition is added when calculating adjusted CPP scores during extinction. The adjusted CPP score derived following the posttest is added to the adjusted CPP score calculated at the end of extinction training. Without the inclusion of the original adjusted CPP score, scores generated following extinction are highly negative, implying aversion to the CS^+^ compartment. During extinction, the subject is not developing an aversion; they are learning that the CS^+^ compartment is no longer associated with the CS^+^. Therefore, adjusted CPP scores generated at the end of extinction training should be close to 0.

ρ_*e*_ and Λ_*e*_ are calculated the same way as depicted in Equations 3 and 4. The subscripts (_1_) and (_2_) now represent the time spent in the CS^+^ compartment during the posttest and at the end of extinction training, respectively. To quantify reinstatement, Equation 2 can be used as normal. The subscripts (_1_) and (_2_) represent the end of extinction training and the reinstatement test, respectively. Figure 7D shows adjusted CPP scores generated at the end of the posttest, the end of extinction training, and the reinstatement test. In agreement with the pre/post difference score analysis, there is a main effect of test period, *F*(2, 44) = 12.232, *p* < 0.001, with adjusted CPP scores being lower following extinction training compared to following the posttest and the reinstatement test. There is also a significant test period × adolescent treatment interaction, *F*(2, 44) = 5.514, *p* = 0.007.

### Applying adjusted CPP scores to conditioned place aversion

The focus of the current paper has been on CPP. However, CPP chambers can be used to measure the conditioned aversive properties of a stimulus; this is conditioned place aversion (CPA). CPA is often achieved by pairing a drug such as lithium to a compartment (Cunningham and Niehus, 1993; Frisch et al., 1995; Longoni et al., 2011; Buffalari et al., 2016) or delivering a foot shock when the animal is in a specific compartment (Buffalari et al., 2016; Barker et al., 2022). CPA is primarily quantified by comparing the time spent in the CS^+^ during the posttest to the time spent in the CS^+^ compartment at pretest (Longoni et al., 2011; Buffalari et al., 2016; Arakaki and Minami, 2022; Peczely et al., 2022; Rezaei et al., 2022), but some studies compare the time spent in the CS^+^ compartment to the time spent in the CS^–^ compartment (Li et al., 2021).

**FIGURE 8:**
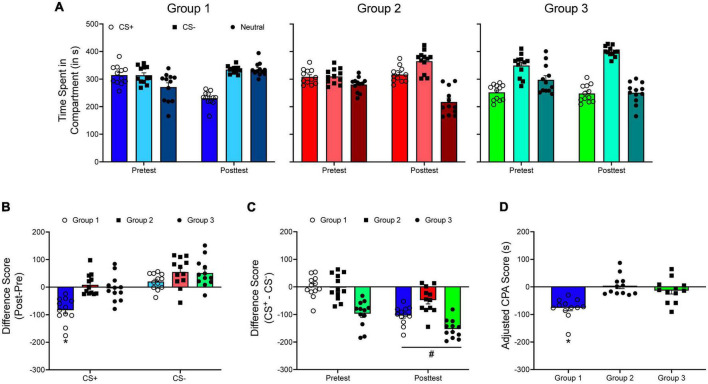
**(A)** The mean (±SEM) number of seconds spent in each compartment of a three-compartment CPP apparatus for three hypothetical groups of animals in a conditioned place aversion (CPA) experiment. **(B)** The mean (±SEM) difference in time spent for each compartment across the pretest and the posttest. *p < 0.05, compared to Groups 2 and 3. **(C)** The mean (±SEM) difference in time spent in the CS^+^ and the CS^–^ compartments during the pretest and during the posttest. #*p* < 0.05, compared to the pretest. **(D)** Mean (±SEM) adjusted CPA scores. *p < 0.05, compared to Groups 2 and 3.

Figure 8A shows three hypothetical groups in a CPA experiment. The data for the first two groups were generated to represent an unbiased design while the data for the third group were generated to represent a biased design. Figure 8B shows posttest/pretest difference scores for both the CS^+^ and the CS^–^ compartments. A one-way ANOVA shows that subjects in the first group develop greater CPA compared to the two other group, *F*(2, 15) = 20.878, *p* < 0.001. However, if difference scores (CS^+^ – CS^–^) are analyzed (Figure 8C), results indicate main effects of test period, *F*(1, 33) = 48.649, *p* < 0.001, and group, *F*(2, 33) = 37.651, *p* < 0.001, but no significant interaction. These results suggest that each group develops a similar degree of CPA.

Like CPP, an adjusted CPA score can be calculated by modifying Equation 2 accordingly:


(4) $$\Psi_{\tau }=\frac{\rho_{\tau }+\Lambda_{\tau }}{2},\mathrm{where}$$

$$\rho_{\tau }=\left(\left({\text{CS}}_{\mathrm{post}}^{+}-{\text{CS}}_{\mathrm{post}}^{-}\right)-\left({\text{CS}}_{\mathrm{pre}}^{+}-{\text{CS}}_{\mathrm{pre}}^{-}\right)\right)$$

$$-\left({\text{Y}}_{\tau }\times \left(1-\left(\frac{{\text{Y}}_{\tau }}{{\text{Y}}_{\tau }+{\text{X}}_{\tau }+{\text{Z}}_{\alpha_{\tau }}+{\text{Z}}_{\beta_{\tau }}}\right)\right)\right)$$

$$\mathrm{and}\Lambda_{\tau }=\left({\text{CS}}_{\mathrm{post}}^{+}-{\text{CS}}_{\mathrm{pre}}^{+}\right)-(\left(\frac{\left({\text{CS}}_{\mathrm{post}}^{+}-{\text{CS}}_{\mathrm{pre}}^{+}\right)-\left|{\text{CS}}_{\mathrm{post}}^{+}-{\text{CS}}_{\mathrm{pre}}^{+}\right|}{2}\right)\times$$

$$\left(1-\left(\frac{\frac{\frac{\left({\text{CS}}_{\mathrm{post}}^{+}-{\text{CS}}_{\mathrm{pre}}^{+}\right)-\left|{\text{CS}}_{\mathrm{post}}^{+}-{\text{CS}}_{\mathrm{pre}}^{+}\right|}{2}}{{\text{CS}}_{\mathrm{pre}}^{+}}}{\begin{array}{c} \frac{\frac{\left({\text{CS}}_{\mathrm{post}}^{+}-{\text{CS}}_{\mathrm{pre}}^{+}\right)-\left|{\text{CS}}_{\mathrm{post}}^{+}-{\text{CS}}_{\mathrm{pre}}^{+}\right|}{2}}{{\text{CS}}_{\mathrm{pre}}^{+}}+\frac{\frac{\left({\text{CS}}_{\mathrm{post}}^{-}-{\text{CS}}_{\mathrm{pre}}^{-}\right)-\left|{\text{CS}}_{\mathrm{post}}^{-}-{\text{CS}}_{\mathrm{pre}}^{-}\right|}{2}}{{\text{CS}}_{\mathrm{pre}}^{-}}\\ +\frac{\frac{\left({\text{N}}_{\mathrm{post}}-{\text{N}}_{\mathrm{pre}}\right)-\left|{\text{N}}_{\mathrm{post}}-{\text{N}}_{\mathrm{pre}}\right|}{2}}{{\text{N}}_{\mathrm{pre}}} \end{array}}\right)\right))$$

Notice that Equation 4 is nearly identical to Equation 2. The major difference is that instead of adding the absolute value of an expression to itself, the absolute value is subtracted. This change applies to the variables Y_τ_, X_τ_, Z_α_τ__, and Z_β_τ__ as well.

When adjusted CPA scores are analyzed (Figure 8D), results are the same as when difference scores (posttest – pretest) are compared across groups: Group 1 develops greater CPA compared to the other groups, *F*(2, 33) = 14.361, *p* < 0.001.

### Determining CPP/CPA in individual subjects

So far, I have presented analyses in which I compare one group of animals to another group of animals. One issue with the CPP/CPA analyses presented so far is that they do not provide information about the expression of CPP/CPA in an *individual* group or in an *individual* subject. Just because one group has a higher CPP score than another group, this does not mean that either group developed CPP.

**FIGURE 9:**
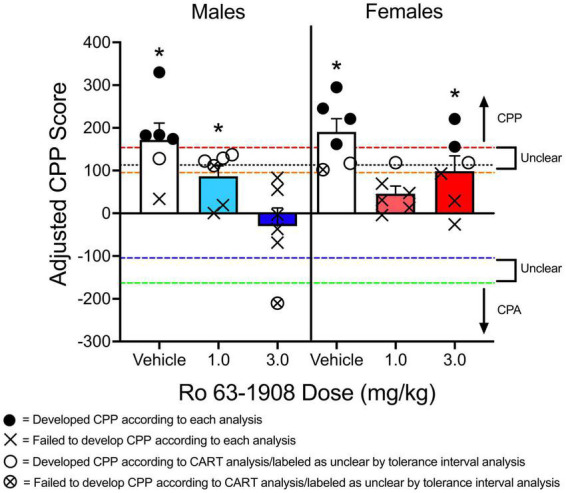
Mean (±SEM) adjusted CPP scores from the experiment described in Figure 4. A one-sample *t* test was used to determine if adjusted CPP scores significantly differ from a value of 0. An * above a group of subjects indicates significant CPP when this analysis is used. A classification and regression tree (CART) analysis was used to identify a critical cut-off value to separate animals as having developed CPP to animals that failed to develop CPP. Animals pretreated with vehicle were compared to control animals that received Ro 63-1908 CPP training (i.e., never received methamphetamine). This analysis identified a value above 113.008 s as meeting CPP (indicated by a black dotted line on the graph). Additionally, [70, 95] and [90, 95] tolerance intervals were built using control animals tested for Ro 63-1908 CPP. This analysis identified CPP-expressing (CPPE) rats as those that had an adjusted CPP score above 153.771 s (denoted by the red dashed line). Subjects with scores between –104.498 s (denoted by the blue dashed line) and 95.166 s (denoted by the orange dashed line) are considered to be CPP-non-expressing (nCPPE). Scores below –163.103 s (green dashed line) denote CPA. Scores between –163.103 s and –104.498 s (between green and blue dashed lines) and between 95.166 and 153.771 s (between orange and red dashed lines) indicates “unclear” rats (i.e., those that cannot be considered CPPE or nCPPE). Closed circles indicate subjects that developed CPP, regardless if the CART analysis or the tolerance interval approach was used. X’s indicate subjects that failed to develop CPP, regardless of analysis type used. Open circles indicate subjects that developed CPP according to the CART analysis, but were classified as unclear according to the tolerance interval analysis. X’s enclosed by a circle indicate subjects that failed to develop CPP according to the CART analysis and were classified as unclear according to the tolerance interval analysis.

One way to determine if a group of animals has developed CPP is to compare CPP scores (raw time in CS^+^ compartment, difference scores, etc.) to a control group that never received the CS^+^ (Ryan et al., 2018; Anooshe et al., 2021; Philogene-Khalid et al., 2022). Because CPP experiments utilize between-subjects designs, a disadvantage to this approach is that additional animals are needed, which can be cost prohibitive. Instead of comparing groups of animals to a control group, some studies have used one-sample *t* tests to compare the mean difference score/preference ratio to a value of 0 (Yates et al., 2012; Aranäs et al., 2021). The one-sample *t* test (or Wilcoxon signed-rank test for non-parametric data) can be used in conjunction with other analyses that directly compare groups to each other. The data from Figure 4D are replotted in Figure 9. Recall that males and females pretreated with vehicle have higher adjusted CPP scores compared to animals pretreated with each dose of Ro 63-1908 (1.0 and 3.0 mg/kg). When a one-sample *t* test is applied to the data, vehicle-pretreated rats show significant CPP, as well as males pretreated with the low dose of Ro 63-1908 and females pretreated with the high dose of Ro 63-1908.

At the individual level, dela Cruz et al. (2009) used a classification and regression tree (CART) analysis to determine a critical “cut-off” value for the expression of cocaine CPP. In this analysis, the time spent in the initially non-preferred compartment was compared between a control group of animals that never received cocaine and an experimental group that learned to associate the initially non-preferred compartment with cocaine. dela Cruz et al. (2009) found 324 s as this criterion score.

As reported by dela Cruz et al. (2009), using CART analysis can lead to different interpretations in a CPP experiment. Testing the effects of the drug MK 212 on cocaine CPP, dela Cruz et al. (2009) found that this drug fails to decrease cocaine CPP when the time spent in the cocaine-paired compartment is compared to a group of rats pretreated with vehicle. However, when rats were categorized as either acquiring CPP or not acquiring CPP, dela Cruz et al. (2009) found that a lower percentage of rats treated with MK 212 (0.125 mg/kg) met the 324-s threshold compared to vehicle-pretreated rats. This is somewhat similar to what is shown in Figure 9 of the current paper. Using the program Orange,^1^ I applied a CART analysis to some of the data presented in Figure 5 of the present paper. Rats pretreated with vehicle before receiving a methamphetamine injection were compared to a control group of rats that did not receive methamphetamine before being conditioned (these rats were tested for Ro 63-1908 CPP). Using this analysis, the cut-off value was >113.008 s. When using this cut-off value, 80% of males and females pretreated with vehicle developed CPP. In contrast, only 1 female pretreated with the low dose of Ro 63-1908 (1.0 mg/kg), and none of the males pretreated with the high dose of Ro 63-1908 (3.0 mg/kg) developed CPP. A chi-square test of independence reveals that the percentage of rats acquiring CPP is dependent on the dose of Ro 63-1908, χ^2^(2, *N* = 36) = 9.585, *p* = 0.008. The proportion of rats expressing CPP was lower for each dose of Ro 63-1908 compared to vehicle-pretreated rats, χ^2^(1, *N* = 24) = 6.171, *p* = 0.013 and χ^2^(1, *N* = 24) = 8.224, *p* = 0.004.

One issue associated with CART analysis is that creating a dichotomous group of subjects based on a single criterion can lead to situations in which a “non-acquiring” subject differs from an “acquiring” subject by just a couple of seconds. This issue is similar to the use of a median split to categorize animals into groups as it artificially dichotomizes continuous data (DeCoster et al., 2011). When examining the data reported in Figure 4C of dela Cruz et al. (2009), there are multiple subjects that are near the cut-off value. This is problematic because the differential results observed in this study seem to be influenced by subjects that have CPP scores near this criterion value. When examining the data, there are *more* subjects treated with MK 212 that spend at least 400 s in the CS^+^ compartment compared to subjects treated with vehicle. However, there are more subjects in this condition that spend less than 300 s in the CS^+^ compartment compared to the vehicle group. In other words, there is less variability in the vehicle-pretreated group relative to the MK 212-pretreated groups. The same issue can be observed in Figure 9 in the current paper. As the cut-off adjusted CPP value was determined to be greater than 113.008 s, there was one male rat treated with the low dose of Ro 63-1908 that developed CPP with a score of 122.331 s but one rat that failed to express CPP with a score of 111.301 s, a difference of just 11.030 s.

Another issue with artificially dichotomizing CPP data is the emergence of ceiling or floor effects in a dataset. Expressing CPP as an “all-or-nothing” event can obscure interesting trends in a dataset. For example, Ro 63-1908 linearly decreases adjusted CPP scores in male rats. That is, most of the males treated with the highest dose of Ro 63-1908 had negative adjusted CPP scores. This linear trend disappears when comparing groups based on a criterion value. Using CART analysis also prevents one from determining if animals pretreated with a pharmacological agent or placed in a particular environmental condition develop CPA as opposed to CPP. For example, one male rat treated with Ro 63-1908 (3.0 mg/kg) had an adjusted CPP score of −210.320 s, indicating a strong aversion to the compartment paired with Ro 63-1908 and methamphetamine.

An alternative approach to determining CPP in individual animals is presented in Atehortua Martinez et al. (2022). A tolerance interval is built using data from a control group of animals that have never receive the CS^+^. The tolerance interval is used to determine a range of values within which a specified proportion of the sampled population falls. Atehortua Martinez et al. (2022) built two tolerance intervals: [70, 95] and [90, 95]. The intervals indicate that one can be 95% confident that either 70% (first interval) or 90% (second interval) of the CPP scores for the control subjects are represented. Results showed that 70% of control subjects should have a CPP score that ranges from −85.1 to 59.4 s and that 90% of control subjects should have CPP scores that range from −128 to 102 s. Animals presented with the CS^+^ (cocaine) are then compared to the intervals generated for the control group. If subjects in the experimental group(s) have CPP scores that fall within the interval of −85 to 59 s, they are classified as non-CPP-expressing (nCPPE). Subjects that have CPP scores above 102 s are considered CPP-expressing (CPPE). If subjects have scores below −85 s or between 59 and 102 s, they are classified as “unclear.” The reason for this is that cocaine animals with a CPP score between −128 and −85 s or between 59 and 102 s were in the same interval as control animals. This makes dissociating these subjects as nCPPE or CPPE difficult. Atehortua Martinez et al. (2022) also characterized rats with scores below −128 s as unclear as they appear to have developed CPA.

Using the same data from the Ro 63-1908/methamphetamine CPP experiment described above, I built tolerance intervals as described in Atehortua Martinez et al. (2022) using the control data reported in Yates et al. (2021a). The [70, 95] interval includes the range of −104.498 s to 95.166 s. The [90, 95] interval includes the range of −163.103 s to 153.771 s. Applying the same protocol as Atehortua Martinez et al. (2022), CPPE rats are those that have an adjusted CPP score of >153.771 s. Rats with a score between −104.498 and 95.166 s are nCPPE. All other rats are considered unclear. Using this approach, four males and four females pretreated with vehicle can be classified as CPPE rats. Only two rats pretreated with Ro 63-1908 (two females pretreated with the high dose) can be classified as CPPE. Figure 9 shows the results of this analysis.

Using tolerance intervals to determine if a subject has developed CPP has the same limitation as CART analysis in that a continuous variable is artificially converted to a categorical variable. However, one advantage of this approach over CART is that it allows one to determine if subjects have developed an aversion to the CS^+^ compartment. While Atehortua Martinez et al. (2022) labeled rats with scores below −128 s as unclear, one could classify these subjects as CPA-expressing (CPAE). If I apply the same logic to the data presented in Figure 9, one male rat pretreated with Ro 63-1908 (3.0 mg/kg) has a score less than −163.103 s, indicating CPA.

Being able to determine if individual subjects have developed CPP/CPA has utility, particularly in studies examining extinction and reinstatement of CPP/CPA. If an animal fails to develop CPP/CPA, there is no need to test them for extinction and reinstatement of CPP/CPA (a behavior that is not established cannot be extinguished). Likewise, determining if an individual subject has met extinction criteria is important as a reinstatement test should not occur until CPP/CPA has been extinguished. Some published studies do not report if they determined if an individual subject developed CPP or met extinction criteria before being tested for reinstatement (Chen Y. et al., 2022; Giacometti et al., 2022; Amirteymori et al., 2023). Instead, one common approach for reinstatement studies is to give subjects a fixed number of extinction sessions before testing them for reinstatement. Other studies set some criteria that need to be met before the reinstatement test can occur (e.g., the time spent in the CS^+^ compartment is similar to the time spent in this compartment during the pretest) (Ferrer-Pérez et al., 2022), but these criteria are set at the group level, not at the individual level.

**FIGURE 10:**
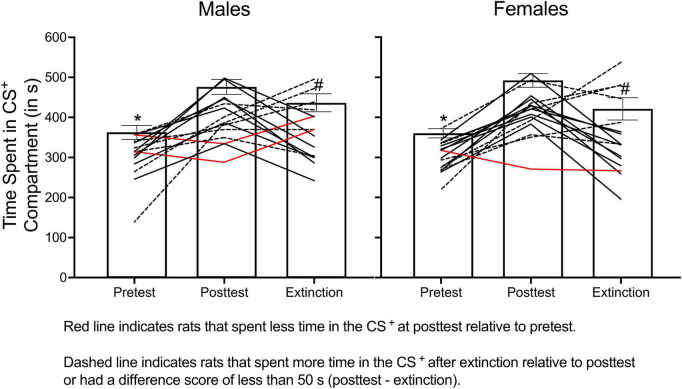
The mean (±SEM) number of seconds spent in the CS^+^ compartment across pretest, posttest, and extinction training for male **(left panel)** and female **(right panel)** rats. When group data are analyzed, results indicate that rats spent less time in the CS^+^ compartment during the pretest compared to the posttest (denoted by *) and spent less time in the CS^+^ compartment after 8 days of extinction training compared to the posttest (denoted by #). When examining individual data points, two males and one female (represented by red lines) spent less time in the CS^+^ compartment at posttest relative to pretest. Six additional males and seven females (represented by dashed lines) either spent more time in the CS^+^ after 8 days of extinction compared to the posttest session or spent marginally less time in this compartment ( < 50 s decrease). While 8 days was selected as the “end” of extinction training for this example, six males and seven females met extinction criteria before 8 extinction sessions; therefore, they did not receive 8 days of extinction training.

Figure 10 shows how relying on group means to determine extinction can be problematic. The data presented in this figure come from a reinstatement experiment conducted in my lab in which we determined if adolescent treatment of methylphenidate increases reinstatement of methamphetamine (1.0 mg/kg) CPP. Some of the data from this experiment are presented in Figure 7. In Figure 10, I present the time spent in the CS^+^ compartment at pretest, at posttest, and at the “end” of extinction training (artificially set at 8 days here) for male and female SHRs. I used 8 days in this example because this is commonly used in CPP experiments measuring extinction and reinstatement (Guzman et al., 2021; Meng et al., 2021; Giacometti et al., 2022; Gonzalez et al., 2022).

A two-way ANOVA was used to determine if the time spent in the CS^+^ compartment changed across each phase of the experiment (pretest vs. posttest vs. extinction) and/or differed across sex. There was no main effect of sex nor a significant interaction. However, the time spent in the CS^+^ compartment changed across each phase of the experiment, *F*(2, 58) = 20.369, *p* < 0.001. Tukey’s *post hoc* test revealed that the time spent in the CS^+^ significantly increased from pretest to posttest and significantly decreased from posttest to extinction. These results suggest that 8 days of extinction training was sufficient to extinguish CPP. When individual data points are examined, only half of the rats showed any evidence of extinction. In our experiment, we found that two males and one female conditioned with methamphetamine (1.0 mg/kg) never acquired CPP (i.e., they spent less time in the CS^+^ compartment at posttest compared to pretest) and that three males and two females never extinguished their preference for the methamphetamine-paired compartment, even after 60 days of extinction training. For the animals that did meet extinction criteria, males needed 12.100 (± 4.092) days to extinguish their preference, and females needed 12.077 (± 3.406) days.

The benefit to using CART analysis or tolerance intervals is that researchers do not need to establish arbitrary criteria for constituting the development of CPP (e.g., 20% increase in time spent in the CS^+^ from pretest to posttest). One disadvantage to these approaches is that a control group of animals is needed to establish the cut-off value/tolerance intervals. Another potential disadvantage of tolerance intervals is attrition. In the Atehortua Martinez et al. (2022) study, they excluded all subjects that were labeled as unclear from further testing, resulting in a loss of 15.3% of the sample. If just trying to determine if CPP has occurred for extinction testing, one could potentially include the subjects that have scores above the upper limit of the [70, 95] tolerance interval instead of labeling them as unclear. Despite these limitations, CART analysis/tolerance intervals can be a great starting point for determining the criteria for the development, the extinction, and the reinstatement of CPP/CPA.

## Discussion

CPP is widely used to study the neurobiological bases of reward (Tzschentke, 2007) and is used to screen potential pharmacotherapies for SUDs (Bardo et al., 2015). Entering the terms “conditioned place preference” OR “place preference” in PubMed, the number of studies published using CPP has increased from one in 1980 (Phillips and LePiane, 1980) to over 250 per year since 2011. There are multiple methodological differences that exist across CPP studies, including the apparatus type used to measure CPP (e.g., two- vs. three-compartment apparatus; biased vs. unbiased), the frequency of conditioning sessions within and across sessions, and the way in which the CS^+^ is assigned to a specific environmental context (e.g., biased vs. unbiased design). Although methodological considerations have been discussed previously (Carr et al., 1989; Cunningham et al., 2003), the purpose of the current paper is to highlight how quantifying CPP can alter one’s interpretation of results, a topic that has not received considerable attention. This topic is important given issues in replicability and transparency in research (Simera et al., 2010; Stevens, 2017). Even if the procedures are replicable, the way in which data are quantified and analyzed can lead to discrepant results across studies.

In the current paper, I proposed the use of the adjusted CPP score to control for potential confounds (e.g., increased novelty seeking) when using a three-compartment apparatus. If a two-compartment apparatus is used, I recommend using an analysis that considers the change in time spent in the CS^+^ compartment from pretest to posttest. Comparing the time spent in the CS^+^ compartment to the time spent in the CS^–^ compartment during the posttest is not ideal as some subjects can spend more time in the CS^+^ compartment relative to the CS^–^ compartment during the pretest. One reason I developed the adjusted CPP score was to provide a way to better standardize how researchers quantify CPP. As detailed earlier in the paper, the same group of researchers using nearly identical methods can derive different conclusions if the way CPP is quantified differs (e.g., Ryan et al., 2018 vs. Randesi et al., 2019). In addition to using these analytic approaches, I encourage researchers to consider using CART analysis or tolerance intervals as a basis for determining if CPP has been established in individual subjects, particularly when extinction and reinstatement of CPP are being examined. This last point is important because many studies measuring reinstatement of CPP often use a fixed number of extinction sessions (see Armstrong et al., 2022; Giacometti et al., 2022; Peeters et al., 2023 for some recent examples). Even if the group average meets extinction criteria, there may be several subjects that have not sufficiently extinguished their CPP. This then can artificially inflate the magnitude of reinstatement.

There are other measures researchers can take to increase transparency and replicability in CPP experiments. First, I recommend the presentation of raw data, specifically the total time spent in each compartment (including the neutral compartment if using a three-compartment apparatus) during the pretest and during the posttest. This will better allow individuals to visualize how the time spent in each compartment changes following conditioning, regardless of which analytic approach is used to quantify CPP. Some journals now encourage or require researchers to upload data to a repository or to include raw data as a supplement. Giving others access to the raw data will improve transparency in research. The raw data used to generate each graph of the present paper is included as a supplement. Second, researchers can present individual data points with the mean scores of each group. Indeed, several journals have adopted policies requiring individuals to include individual data points on figures. This, in conjunction with CART analysis/tolerance intervals, can help readers identify which individual subjects developed CPP/CPA. Although not directly related to data analysis, a final step that can be taken to ensure replicability and transparency is to clearly include the following information in the methods section of a paper: number of compartments in the apparatus, if the apparatus is biased or unbiased, and if the researchers used a biased or unbiased design. Collectively, these measures can help ensure that others can replicate a CPP/CPA experiment.

## Data availability statement

The original contributions presented in this study are included in the article/Supplementary material; further inquiries can be directed to the corresponding author.

## Author contributions

JY: Data curation, Formal analysis, Methodology, Visualization, Writing – original draft, Writing – review & editing, and conceptualization.

## Footnotes

1
https://orangedatamining.com/widget-catalog/model/tree/

## References

[B1] Amirteymori H. Veisi A. Khaleghzadeh-Ahangar H. Mozafari R. Haghparast A. (2023). Involvement of orexin-2 receptors in the CA1 region of the hippocampus in the extinction and reinstatement of methamphetamine-induced conditioned place preference in the rats. *Peptides* 160:170926. 10.1016/j.peptides.2022.170926 36565856

[B2] Anooshe M. Nouri K. Karimi-Haghighi S. Mousavi Z. Haghparast A. (2021). Cannabidiol efficiently suppressed the acquisition and expression of methamphetamine-induced conditioned place preference in the rat. *Behav. Brain Res.* 404:113158. 10.1016/j.bbr.2021.113158 33571569

[B3] Arakaki S. Minami M. (2022). Role of noradrenergic transmission within the ventral bed nucleus of the stria terminalis in nicotine withdrawal-induced aversive behavior. *Neuropsychopharmacol. Rep.* 42 233–237. 10.1002/npr2.12252 35437943 PMC9216371

[B4] Aranäs C. Vestlund J. Witley S. Edvardsson C. E. Kalafateli A. L. Jerlhag E. (2021). Salmon calcitonin attenuates some behavioural responses to nicotine in male mice. *Front. Pharmacol.* 12:685631. 10.3389/fphar.2021.685631 34234676 PMC8257032

[B5] Armstrong C. Ferrante J. Lamichhane N. Reavis Z. Walker D. Patkar A. (2022). Rapastinel accelerates loss of withdrawal signs after repeated morphine and blunts relapse to conditioned place preference. *Pharmacol. Biochem. Behav.* 221:173485. 10.1016/j.pbb.2022.173485 36302442

[B6] Atehortua Martinez L. A. Curis E. Mekdad N. Larrieu C. Courtin C. Jourdren L. (2022). Individual differences in cocaine-induced conditioned place preference in male rats: Behavioral and transcriptomic evidence. *J. Psychopharmacol.* 36 1161–1175. 10.1177/02698811221123047 36121009 PMC9548661

[B7] Avelar A. J. Cooper S. Y. Wright T. D. Wright S. K. Richardson M. R. Henderson B. J. (2022). Morphine exposure reduces nicotine-induced upregulation of nicotinic receptors and decreases volitional nicotine intake in a mouse model. *Nicotine Tob. Res.* 24 1161–1168. 10.1093/ntr/ntac002 34999827 PMC9278828

[B8] Bardo M. T. Bevins R. A. (2000). Conditioned place preference: What does it add to our preclinical understanding of drug reward? *Psychopharmacology* 153 31–43. 10.1007/s002130000569 11255927

[B9] Bardo M. T. Horton D. B. Yates J. R. (2015). “Conditioned place preference as a preclinical model for screening pharmacotherapies for drug abuse,” in *Nonclinical assessment of abuse potential for new pharmacotherapies*, eds Markgraf C. G. Hudzik T. J. Comptom D. R. (London: Academic Press), 151–196.

[B10] Barker A. M. Moore H. N. Buffalari D. (2022). Sex differences in nicotine enhancement of conditioned place avoidance driven by footshock in male and female rats. *Nicotine Tob. Res.* 24 1689–1692. 10.1093/ntr/ntac109 35439811

[B11] Bowling S. L. Bardo M. T. (1994). Locomotor and rewarding effects of amphetamine in enriched, social, and isolate reared rats. *Pharmacol. Biochem. Behav.* 48 459–464. 10.1016/0091-3057(94)90553-3 8090815

[B12] Buffalari D. M. Mollica J. K. Smith T. T. Schassburger R. L. Rinaman L. Thiels E. (2016). Nicotine enhances footshock- and lithium chloride-conditioned place avoidance in male rats. *Nicotine Tob. Res.* 18 1920–1923. 10.1093/ntr/ntw098 27178831 PMC4978983

[B13] Calcagnetti D. J. Schechter M. D. (1992). Place conditioning reveals the rewarding aspect of social interaction in juvenile rats. *Physiol. Behav.* 51 667–672. 10.1016/0031-9384(92)90101-7 1594664

[B14] Cann C. Venniro M. Hope B. T. Ramsey L. A. (2020). Parametric investigation of social place preference in adolescent mice. *Behav. Neurosci.* 134 435–443. 10.1037/bne0000406 32672990 PMC8189642

[B15] Carmack S. A. Kim J. S. Sage J. R. Thomas A. W. Skillicorn K. N. Anagnostaras S. G. (2013). The competitive NMDA receptor antagonist CPP disrupts cocaine-induced conditioned place preference, but spares behavioral sensitization. *Behav. Brain Res.* 239 155–163. 10.1016/j.bbr.2012.10.042 23153931

[B16] Carr G. D. Fibiger H. C. Phillips A. G. (1989). “Conditioned place preference as a measure of drug reward,” in *The Neuropharmacological Basis of Reward*, eds Liebman J. M. Cooper S. J. (Oxford: Oxford University Press), 264–319.

[B17] Chen H. Chen L. Yuan Z. Yuan J. Li Y. Xu Y. (2022). Glutamate receptor-interacting protein 1 in D1- and D2-dopamine receptor-expressing medium spiny neurons differentially regulates cocaine acquisition, reinstatement, and associated spine plasticity. *Front. Cell Neurosci.* 16:979078. 10.3389/fncel.2022.979078 36406750 PMC9669444

[B18] Chen Y. Zhang L. Ding Z. Wu X. Wang G. Shi J. (2022). Effects of 3-methylmethcathinone on conditioned place preference and anxiety-like behavior: Comparison with methamphetamine. *Front. Mol. Neurosci.* 15:975820. 10.3389/fnmol.2022.975820 35935336 PMC9354685

[B19] Chen H. Xu D. Zhang Y. Yan Y. Liu J. Liu C. (2021). Neurons in the locus coeruleus modulate the hedonic effects of sub-anesthetic dose of propofol. *Front. Neurosci.* 15:636901. 10.3389/fnins.2021.636901 33767609 PMC7985178

[B20] Cicero T. J. Ennis T. Ogden J. Meyer E. R. (2000). Gender differences in the reinforcing properties of morphine. *Pharmacol. Biochem. Behav.* 65 91–96. 10.1016/s0091-3057(99)00174-4 10638641

[B21] Cooper S. Y. Akers A. T. Journigan V. B. Henderson B. J. (2021). Novel putative positive modulators of α4β2 nAChRs potentiate nicotine reward-related behavior. *Molecules* 26:4793. 10.3390/molecules26164793 34443380 PMC8398432

[B22] Cunningham C. L. Ferree N. K. Howard M. A. (2003). Apparatus bias and place conditioning with ethanol in mice. *Psychopharmacology* 170 409–422. 10.1007/s00213-003-1559-y 12955296

[B23] Cunningham C. L. Niehus J. S. (1993). Drug-induced hypothermia and conditioned place aversion. *Behav. Neurosci.* 107 468–479. 10.1037//0735-7044.107.3.468 8329136

[B24] de Vries T. J. Babovic-Vuksanovic D. Elmer G. Shippenberg T. S. (1995). Lack of involvement of delta-opioid receptors in mediating the rewarding effects of cocaine. *Psychopharmacology* 120 442–448. 10.1007/BF02245816 8539325

[B25] DeCoster J. Gallucci M. Iselin A.-M. R. (2011). Best practices for using median splits, artificial categorization, and their continuous alternatives. *J. Exp. Psychopathol.* 2 197–209. 10.5127/jep.008310

[B26] dela Cruz A. M. Herin D. V. Grady J. J. Cunningham K. A. (2009). Novel approach to data analysis in cocaine-conditioned place preference. *Behav. Pharmacol.* 20 720–730. 10.1097/FBP.0b013e328333b266 19901823 PMC3725613

[B27] Duarte C. Lefebvre C. Chaperon F. Hamon M. Thiébot M. H. (2003). Effects of a dopamine D3 receptor ligand, BP 897, on acquisition and expression of food-, morphine-, and cocaine-induced conditioned place preference, and food-seeking behavior in rats. *Neuropsychopharmacology* 28 1903–1915. 10.1038/sj.npp.1300276 12915863

[B28] Ewin S. E. Kangiser M. M. Stairs D. J. (2015). The effects of environmental enrichment on nicotine condition place preference in male rats. *Exp. Clin. Psychopharmacol.* 23 387–394. 10.1037/pha0000024 26167715

[B29] Ferrer-Pérez C. Reguilón M. D. Miñarro J. Rodríguez-Arias M. (2022). Effect of voluntary wheel-running exercise on the endocrine and inflammatory response to social stress: Conditioned rewarding effects of cocaine. *Biomedicines* 10:2373. 10.3390/biomedicines10102373 36289635 PMC9598819

[B30] Frisch C. Hasenöhrl R. U. Mattern C. M. Häcker R. Huston J. P. (1995). Blockade of lithium chloride-induced conditioned place aversion as a test for antiemetic agents: Comparison of metoclopramide with combined extracts of *Zingiber officinale* and *Ginkgo biloba*. *Pharmacol. Biochem. Behav.* 52 321–327. 10.1016/0091-3057(95)00073-6 8577797

[B31] Gargiulo A. T. Badve P. S. Curtis G. R. Prino B. E. Barson J. R. (2022). Inactivation of the thalamic paraventricular nucleus promotes place preference and sucrose seeking in male rats. *Psychopharmacology* 239 2659–2671. 10.1007/s00213-022-06160-2 35524009 PMC9296579

[B32] Giacometti L. L. Buck L. A. Barker J. M. (2022). Estrous cycle and hormone regulation of stress-induced reinstatement of reward seeking in female mice. *Addict. Neurosci.* 4:100035. 10.1016/j.addicn.2022.100035 36540408 PMC9762733

[B33] Gonzalez A. E. Jorgensen E. T. Ramos J. D. Harkness J. H. Aadland J. A. Brown T. E. (2022). Impact of perineuronal net removal in the rat medial prefrontal cortex on parvalbumin interneurons after reinstatement of cocaine conditioned place preference. *Front. Cell Neurosci.* 16:932391. 10.3389/fncel.2022.932391 35966203 PMC9366391

[B34] Guterl S. A. McNamara T. A. Klumpp G. C. Meerts S. H. (2015). Female rats express a conditioned object preference for receipt of sexual stimulation. *Physiol. Behav.* 151 320–326. 10.1016/j.physbeh.2015.07.040 26247393

[B35] Guzman A. S. Avalos M. P. De Giovanni L. N. Euliarte P. V. Sanchez M. A. Mongi-Bragato B. (2021). CB1R activation in nucleus accumbens core promotes stress-induced reinstatement of cocaine seeking by elevating extracellular glutamate in a drug-paired context. *Sci. Rep.* 11:12964. 10.1038/s41598-021-92389-4 34155271 PMC8217548

[B36] Hachimine P. Seepersad N. Ananthan S. Ranaldi R. (2014). The novel dopamine D3 receptor antagonist, SR 21502, reduces cocaine conditioned place preference in rats. *Neurosci. Lett.* 569 137–141. 10.1016/j.neulet.2014.03.055 24704326

[B37] Hayes D. J. Mosher T. M. Greenshaw A. J. (2009). Differential effects of 5-HT2C receptor activation by WAY 161503 on nicotine-induced place conditioning and locomotor activity in rats. *Behav. Brain Res.* 197 323–330. 10.1016/j.bbr.2008.08.034 18805442

[B38] Huerta C. Paredes R. G. Morales T. Daniel Caba-Flores M. Meza E. Caba M. (2022). Rabbits can be conditioned in a food-induced place preference paradigm. *Brain Res.* 1781:147815. 10.1016/j.brainres.2022.147815 35131285

[B39] Ismail N. Laroche C. Girard-Bériault F. Ménard S. Greggain-Mohr J. A. Pfaus J. G. (2010). Conditioned ejaculatory preference in male rats paired with haloperidol-treated females. *Physiol. Behav.* 100 116–121. 10.1016/j.physbeh.2010.02.007 20159027

[B40] Jamali S. Zarrabian S. Haghparast A. (2021). Similar role of mPFC orexin-1 receptors in the acquisition and expression of morphine- and food-induced conditioned place preference in male rats. *Neuropharmacology* 198:108764. 10.1016/j.neuropharm.2021.108764 34450116

[B41] Karami M. Zarrindast M. R. (2008). Morphine sex-dependently induced place conditioning in adult Wistar rats. *Eur. J. Pharmacol.* 582 78–87. 10.1016/j.ejphar.2007.12.010 18191832

[B42] Kitanaka N. Kitanaka J. Watabe K. Takemura M. (2010). Low-dose pretreatment with clorgyline decreases the levels of 3-methoxy-4-hydroxyphenylglycol in the striatum and nucleus accumbens and attenuates methamphetamine-induced conditioned place preference in rats. *Neuroscience* 165 1370–1376. 10.1016/j.neuroscience.2009.11.058 19958817

[B43] Kosten T. A. Miserendino M. J. Chi S. Nestler E. J. (1994). Fischer and Lewis rat strains show differential cocaine effects in conditioned place preference and behavioral sensitization but not in locomotor activity or conditioned taste aversion. *J. Pharmacol. Exp. Ther.* 269 137–144. 8169817

[B44] Kummer K. K. Hofhansel L. Barwitz C. M. Schardl A. Prast J. M. Salti A. (2014). Differences in social interaction- vs. cocaine reward in mouse vs. rat. *Front. Behav. Neurosci.* 8:363. 10.3389/fnbeh.2014.00363 25368560 PMC4201146

[B45] Li X. Hempel B. J. Yang H. J. Han X. Bi G. H. Gardner E. L. (2021). Dissecting the role of CB_1_ and CB_2_ receptors in cannabinoid reward versus aversion using transgenic CB_1_- and CB_2_-knockout mice. *Eur. Neuropsychopharmacol.* 43 38–51. 10.1016/j.euroneuro.2020.11.019 33334652 PMC7854511

[B46] Longoni R. Spina L. Vinci S. Acquas E. (2011). The MEK inhibitor SL327 blocks acquisition but not expression of lithium-induced conditioned place aversion: A behavioral and immunohistochemical study. *Psychopharmacology* 216 63–73. 10.1007/s00213-011-2192-9 21312031

[B47] Luo J. Bian L. H. Yao Z. W. Wang X. M. Li Q. Y. Guo J. Y. (2021). Anthocyanins in Lycium ruthenicum Murray reduce nicotine withdrawal-induced anxiety and craving in mice. *Neurosci. Lett.* 763:136152. 10.1016/j.neulet.2021.136152 34384845

[B48] Ma Y. Y. Guo C. Y. Yu P. Lee D. Y. Han J. S. Cui C. L. (2006). The role of NR2B containing NMDA receptor in place preference conditioned with morphine and natural reinforcers in rats. *Exp. Neurol.* 200 343–355. 10.1016/j.expneurol.2006.02.117 16631172

[B49] McKendrick G. Graziane N. M. (2020). Drug-induced conditioned place preference and its practical use in substance use disorder research. *Front. Behav. Neurosci.* 14:582147. 10.3389/fnbeh.2020.582147 33132862 PMC7550834

[B50] Meerts S. H. Clark A. S. (2007). Female rats exhibit a conditioned place preference for nonpaced mating. *Horm. Behav.* 51 89–94. 10.1016/j.yhbeh.2006.08.007 17020761

[B51] Meng S. Yan W. Liu X. Gong Y. Tian S. Wu P. (2021). Social interaction with relapsed partner facilitates cocaine relapse in rats. *Front. Pharmacol.* 12:750397. 10.3389/fphar.2021.750397 34671262 PMC8520921

[B52] Menkens K. Bilsky E. J. Wild K. D. Portoghese P. S. Reid L. D. Porreca F. (1992). Cocaine place preference is blocked by the delta-opioid receptor antagonist, naltrindole. *Eur. J. Pharmacol.* 219 345–346. 10.1016/0014-2999(92)90319-y 1425962

[B53] Nesbit M. O. Dias C. Phillips A. G. (2017). The effects of d-govadine on conditioned place preference with d-amphetamine or food reward. *Behav. Brain Res.* 321 223–231. 10.1016/j.bbr.2016.12.043 28062254

[B54] Peczely L. Ollmann T. Laszlo K. Lenard L. Grace A. A. (2022). The D2-like dopamine receptor agonist quinpirole microinjected into the ventral pallidum dose-dependently inhibits the VTA and induces place aversion. *Int. J. Neuropsychopharmacol.* 25 590–599. 10.1093/ijnp/pyac024 35348731 PMC9352176

[B55] Peeters L. D. Wills L. J. Cuozzo A. M. Ivanich K. L. Brown R. W. (2023). Reinstatement of nicotine conditioned place preference in a transgenerational model of drug abuse vulnerability psychosis: Impact of BDNF on the saliency of drug associations. *Psychopharmacology* 240 1453–1464. 10.1007/s00213-023-06379-7 37160431 PMC10330905

[B56] Phillips A. G. LePiane F. G. (1980). Reinforcing effects of morphine microinjection into the ventral tegmental area. *Pharmacol. Biochem Behav.* 12 965–968. 10.1016/0091-3057(80)90460-8 7403209

[B57] Philogene-Khalid H. L. Morrison M. F. Darbinian N. Selzer M. E. Schroeder J. Rawls S. M. (2022). The GLT-1 enhancer clavulanic acid suppresses cocaine place preference behavior and reduces GCPII activity and protein levels in the rat nucleus accumbens. *Drug Alcohol Depend.* 232:109306. 10.1016/j.drugalcdep.2022.109306 35051699 PMC8885893

[B58] Pinheiro B. S. Seidl S. S. Habazettl E. Gruber B. E. Bregolin T. Zernig G. (2016). Dyadic social interaction of C57BL/6 mice versus interaction with a toy mouse: Conditioned place preference/aversion, substrain differences, and no development of a hierarchy. *Behav. Pharmacol.* 27 279–288. 10.1097/FBP.0000000000000223 26905190 PMC4780246

[B59] Poleszak E. Malec D. (2002). Adenosine receptor ligands and cocaine in conditioned place preference (CPP) test in rats. *Pol. J. Pharmacol.* 54 119–126. 12139108

[B60] Quintana G. R. González B. Borduas E. Lemay V. Yarur F. Pfaus J. G. (2019). Naloxone disrupts the development of a conditioned ejaculatory preference based on a somatosensory cue in male rats. *Behav. Neurosci.* 133 198–202. 10.1037/bne0000302 30714805

[B61] Randesi M. Contoreggi N. H. Zhou Y. Reich B. Bellamy J. R. Yu F. (2019). Sex differences in neuroplasticity- and stress-related gene expression and protein levels in the rat hippocampus following oxycodone conditioned place preference. *Neuroscience* 410 274–292. 10.1016/j.neuroscience.2019.04.047 31071414 PMC7746310

[B62] Rezaei Z. Alaei H. Reisi P. (2022). Involvement of basolateral amygdala dopamine D1 receptors in the acquisition and expression of morphine-induced place preference in rats. *Adv. Biomed. Res.* 11:8. 10.4103/abr.abr_284_21 35284350 PMC8906092

[B63] Richardson J. R. O’Dell L. E. Nazarian A. (2020). Examination of nicotine and saccharin reward in the Goto-Kakizaki diabetic rat model. *Neurosci. Lett.* 721:134825. 10.1016/j.neulet.2020.134825 32036029 PMC7043340

[B64] Ryan J. D. Zhou Y. Contoreggi N. H. Bshesh F. K. Gray J. D. Kogan J. F. (2018). Sex differences in the rat hippocampal opioid system after oxycodone conditioned place preference. *Neuroscience* 393 236–257. 10.1016/j.neuroscience.2018.10.002 30316908 PMC6246823

[B65] Simera I. Moher D. Hirst A. Hoey J. Schulz K. F. Altman D. G. (2010). Transparent and accurate reporting increases reliability, utility, and impact of your research: Reporting guidelines and the EQUATOR Network. *BMC Med.* 8:24. 10.1186/1741-7015-8-24 20420659 PMC2874506

[B66] Stevens J. R. (2017). Replicability and reproducibility in comparative psychology. *Front. Psychol.* 8:862. 10.3389/fpsyg.2017.00862 28603511 PMC5445189

[B67] Stojakovic A. Ahmad S. M. Lutfy K. (2021). Alterations of amphetamine reward by prior nicotine and alcohol treatment: The role of age and dopamine. *Brain Sci.* 11:420. 10.3390/brainsci11040420 33810331 PMC8065622

[B68] Trezza V. Damsteegt R. Vanderschuren L. J. (2009). Conditioned place preference induced by social play behavior: Parametrics, extinction, reinstatement and disruption by methylphenidate. *Eur. Neuropsychopharmacol.* 19 659–669. 10.1016/j.euroneuro.2009.03.006 19427175 PMC2716414

[B69] Tzschentke T. M. (1998). Measuring reward with the conditioned place preference paradigm: A comprehensive review of drug effects, recent progress and new issues. *Prog. Neurobiol.* 56 613–672. 10.1016/s0301-0082(98)00060-4 9871940

[B70] Tzschentke T. M. (2007). Measuring reward with the conditioned place preference (CPP) paradigm: Update of the last decade. *Addict. Biol.* 12 227–462. 10.1111/j.1369-1600.2007.00070.x 17678505

[B71] Velazquez-Sanchez C. Ferragud A. Renau-Piqueras J. Canales J. J. (2011). Therapeutic-like properties of a dopamine uptake inhibitor in animal models of amphetamine addiction. *Int. J. Neuropsychopharmacol.* 14 655–665. 10.1017/S1461145710000969 20735880

[B72] Watterson E. Daniels C. W. Watterson L. R. Mazur G. J. Brackney R. J. Olive M. F. (2015). Nicotine-induced place conditioning and locomotor activity in an adolescent animal model of attention deficit/hyperactivity disorder (ADHD). *Behav. Brain Res.* 291 184–188. 10.1016/j.bbr.2015.05.031 26008156 PMC4497919

[B73] Weitz M. Khayat A. Yaka R. (2021). GABAergic projections to the ventral tegmental area govern cocaine-conditioned reward. *Addict. Biol.* 2021:e13026. 10.1111/adb.13026 33638301

[B74] Yates J. R. Beckmann J. S. Meyer A. C. Bardo M. T. (2013). Concurrent choice for social interaction and amphetamine using conditioned place preference in rats: Effects of age and housing condition. *Drug Alcohol Depend.* 129 240–246. 10.1016/j.drugalcdep.2013.02.024 23540449 PMC3628407

[B75] Yates J. R. Campbell H. L. Hawley L. L. Horchar M. J. Kappesser J. L. Wright M. R. (2021a). Effects of the GluN2B-selective antagonist Ro 63-1908 on acquisition and expression of methamphetamine conditioned place preference in male and female rats. *Drug Alcohol Depend.* 225:108785. 10.1016/j.drugalcdep.2021.108785 34052688 PMC8282733

[B76] Yates J. R. Horchar M. J. Kappesser J. L. Broderick M. R. Ellis A. L. Wright M. R. (2021b). The association between risky decision making and cocaine conditioned place preference is moderated by sex. *Drug Alcohol Depend.* 228:109079. 10.1016/j.drugalcdep.2021.109079 34600260 PMC8595855

[B77] Yates J. R. Marusich J. A. Gipson C. D. Beckmann J. S. Bardo M. T. (2012). High impulsivity in rats predicts amphetamine conditioned place preference. *Pharmacol. Biochem. Behav.* 100 370–376. 10.1016/j.pbb.2011.07.012 21807020 PMC3242916

[B78] Zernig G. Kummer K. K. Prast J. M. (2013). Dyadic social interaction as an alternative reward to cocaine. *Front. Psychiatry* 4:100. 10.3389/fpsyt.2013.00100 24062696 PMC3770939

[B79] Zhao Y. Liu P. Chu Z. Liu F. Han W. Xun X. (2015). Electrolytic lesions of the bilateral ventrolateral orbital cortex inhibit methamphetamine-associated contextual memory formation in rats. *Brain Res.* 1624 214–221. 26248279 10.1016/j.brainres.2015.07.046

