# Supplementary material for: Quantifying conditioned place preference: a review of current analyses and a proposal for a novel approach
Source: Front Behav Neurosci. 2023 Aug 24;17:1256764. doi: 10.3389/fnbeh.2023.1256764 (PMC10484009; doi:10.3389/fnbeh.2023.1256764)
Supplement: Supplementary file 1 [file Table_1.docx]

| **Supplementary Table 1**  Analytic approaches used in stimulant CPP research published from January 2021 until December 2022.   \| **CS^+^** \| **Species** \| **Design** \| **Number of Compartments** \| **How CPP was Operationalized** \| **Citation(s)** \| \| --- \| --- \| --- \| --- \| --- \| --- \| \| AMPH \| Rat \| Biased \| 3 \| % time spent in CS^+^ during posttest compared to control group never treated with AMPH \| Metz et al. (2021; 2022); Rossato et al. (2022)^a^; Segat et al. (2022) \| \|  \|  \|  \|  \| Raw time spent in CS^+^ across pretest and posttest \| Gárate-Pérez et al. (2021) \| \|  \|  \|  \|  \| Raw time spent in CS^+^ and CS^-^ \| [Sánchez-Zavaleta](https://pubmed.ncbi.nlm.nih.gov/?sort=date&size=200&term=S%C3%A1nchez-Zavaleta+R&cauthor_id=36099968) et al. (2023) \| \|  \|  \|  \|  \| Difference score (post – pre) \| Elgueta-Reyes et al. (2022) \| \|  \|  \|  \|  \| Difference score (CS^+^ – CS^-^) for both pretest and posttest \| Migliaro et al. (2022) \| \|  \|  \|  \| 2 \| Difference score (post – pre) \| Hilz et al. (2022) \| \|  \| Mouse \|  \| 3 \| Difference score (post – pre) \| Mannangatti et al. (2021); Ragu Varman et al. (2022) \| \|  \|  \|  \| 1^b^ \| Ratio calculated as [time spent on cocaine-paired floor (at posttest) – time spent on cocaine-paired floor (at pretest)] / [total test time] * 100 \| Shetty et al. (2022) \| \| AMPH \| Mouse \| Unbiased \| 3 \| Raw time spent in CS^+^ and CS^-^ \| Stojakovic et al. (2021) \| \|  \|  \|  \| 2 \| Difference score (post – pre) \| Babigian et al. (2022) \| \|  \|  \|  \|  \| Raw time spent in CS^+^ and CS^-^ \| Valenti et al. (2021) \| \|  \|  \|  \| 1^b^ \| Difference score (CS^+^ – CS^-^) for posttest only \| Clauss et al. (2021) \| \| Cocaine \| Rat \| Both^c^ \| 3 \| Raw time spent in CS^+^ across pretest and posttest \| Gonzalez et al. (2022) \| \|  \|  \|  \| 2 \| % time spent in CS^+^ across pretest and posttest \| Guerin et al. (2021) \| \|  \|  \|  \| ?^d^ \| % time spent in CS^+^ across pretest and posttest \| Nedelescu et al. (2022) \| \|  \|  \| Biased \| 3 \| Difference score (post – pre) \| Lemos et al. (2021); [Kołosowska](https://pubmed.ncbi.nlm.nih.gov/?term=Ko%C5%82osowska%20K%5BAuthor%5D) et al. (2023) \| \|  \|  \|  \|  \| Raw time spent in CS^+^ across pretest and posttest \| Pastor et al. (2021); Quigley and Becker (2021); Yates et al. (2021b) \| \|  \|  \|  \|  \| Raw time spent in CS^+^ during posttest only \| Amaral et al. (2021); [Barbosa-Méndez](https://pubmed.ncbi.nlm.nih.gov/?sort=date&size=200&term=Barbosa-M%C3%A9ndez+S&cauthor_id=33080430) et al. (2021) \| \|  \|  \|  \|  \| Ratio calculated as 100*((CS^+^_post_ - CS^-^_post_)/(CS^+^_post_ + CS^-^_post_)) \| Weitz et al. (2021) \| \| Cocaine \| Rat \| Biased \| 2 \| Difference score (post – pre) \| Philogene-Khalid et al. (2022) \| \|  \|  \|  \|  \| Difference score (CS^+^ – CS^-^) for both pretest and posttest \| Guzman et al. (2021) \| \|  \|  \| Unbiased \| 3 \| Difference score (post – pre) \| Caffino et al. (2021); Rohan et al. (2021); Atehortua Martinez et al. (2022) \| \|  \|  \|  \|  \| Difference score (CS+ – CS-) for both pretest and posttest \| Meng et al. (2021) \| \|  \|  \|  \|  \| Difference score (CS^+^ - CS^-^)_post_ – (CS^+^ - CS^-^)_pre_ \| Parrilla-Carrero et al. (2021) \| \|  \|  \|  \|  \| Ratio calculated as CS^+^/(CS^+^ + CS^-^) \| Hammad et al. (2021) \| \|  \|  \|  \|  \| Raw time spent in CS^+^ across pretest and posttest \| Galaj et al. (2021); Shaw et al. (2021) \| \|  \| Mouse \| Both^c^ \| 3 \| Ratio calculated as 100*((CS^+^_post_ - CS^-^_post_)/(CS^+^_post_ + CS^-^_post_)) \| Mañas-Padilla et al. (2021) \| \|  \|  \|  \| 2 \| Raw time spent in CS^+^ across pretest and posttest \| Gomez et al. (2021); de Guglielmo et al. (2022) \| \|  \|  \| Biased \| 3 \| Difference score (post – pre) \| Xu et al. (2021); Abraham et al. (2022) \| \| Cocaine \| Mouse \| Biased \| 3 \| Difference score (CS+ – CS-) for both pretest and posttest \| Chang et al. (2021); Chou et al. (2021); Chang et al. (2022) \| \|  \|  \|  \|  \| Raw time spent in CS^+^ across pretest and posttest \| Zhu C. et al. (2021a; 2021b); Zhu L. et al. (2022) \| \|  \|  \|  \|  \| % time spent in CS^+^ across pretest and posttest \| Zipperly et al. (2021) \| \|  \|  \|  \|  \| Ratio calculated as 100*((CS^+^_post_ - CS^-^_post_)/(CS^+^_post_ + CS^-^_post_)) \| Beiser et al. (2022); Shahen-Zoabi et al. (in press) \| \|  \|  \|  \|  \| Ratio calculated as 100*((CS^+^)/(CS^+^ + CS^-^)) \| Li W. et al. (2022) \| \|  \|  \| Biased \| 2 \| Difference score (post – pre) \| Chu et al. (2021); Preston and Wagner (2022) \| \|  \|  \|  \|  \| Difference score (CS+ – CS-) for both pretest and posttest \| Jiang et al. (2021); Xue et al. (2021) \| \|  \|  \|  \|  \| Raw time spent in CS^+^ across pretest and posttest \| Nguyen et al. (2021); Turner et al. (2021); Zhao et al. (2022) \| \|  \|  \|  \|  \| % difference in time spent in CS^+^ across pretest and posttest \| Kalafateli et al. (2021) \| \|  \|  \|  \|  \| % side preference across pretest and posttests^f^ \| Siemian et al. (2021) \| \| Cocaine \| Mouse \| Unbiased \| 3 and 2 \| Difference score (post – pre) \| Rosa et al. (2022) \| \|  \|  \|  \| 3 \| Difference score (post – pre) \| Asth et al. (2021); Ballestín et al. (2021); Ródenas-González et al. (2021a); Sutton et al. (2021); Calpe-López et al. (2022a); Fontaine et al. (2022); Li H. et al. (2022); Reguilón et al. (2022); Zhu J. et al. (2022); Darcq et al. (2023) \| \|  \|  \|  \|  \| Difference score (CS+ – CS-) for both pretest and posttest \| Gobira et al. (2021); Harraz et al. (2021); Visser et al. (2022); Wang Y. et al. (2023) \| \|  \|  \|  \|  \| Difference score (CS+ – CS-) for posttest only \| Li J. et al. (2023a) \| \|  \|  \|  \|  \| Raw time spent in CS^+^ across pretest and posttest \| Calpe-López et al. (2021); Christoffel et al. (2021); da Silva et al. (2021); Fugazzotto et al. (2021); Giménez-Gómez et al. (2021); Guerrero-Bautista et al. (2021); Jorgensen et al. (2021); Ledesma et al. (2021); Melchior et al. (2021); Montagud-Romero et al. (2021); Pujante-Gil et al. (2021); Ródenas-González et al. (2021b); Singh and Lutfy (2021)^g^; Arenäs et al. (2022); Calpe-López et al. (2022b); Ferrer-Pérez et al. (2022); Reguilón et al. (2022); Ródenas-González et al. (2022); Martínez-Rivera et al. (2023) \| \| Cocaine \| Mouse \| Unbiased \| 3 \| Time spent in CS^+^ × total time/(total time – time in neutral compartment) normalized to baseline \| Panopoulou and Schlüter (2022) \| \|  \|  \|  \| 2 \| Difference score (post – pre) \| Hillhouse et al. (2021); Kawahara et al. (2021); Singh et al. (2022) \| \|  \|  \|  \|  \| Difference score (CS^+^ – CS^-^) posttest only \| Shi et al. (2022) \| \|  \|  \|  \|  \| % time spent in CS^+^ across pretest and posttest \| Kähler et al. (2021); Dai et al. (2022) \| \|  \|  \|  \|  \| Ratio calculated as (CS^+^_post_ - CS^-^_post_)/(CS^+^_post_ + CS^-^_post_) \| Inbar et al. (2022) \| \|  \|  \|  \| 1^b^ \| % time spent in CS^+^ across pretest and posttest \| Mukherjee et al. (2021) \| \|  \|  \|  \|  \| Ratio calculated as (CS^+^_post_/(CS^+^_post_ + CS^-^_post_)) - (CS^+^_pre_/(CS^+^_pre_ + CS^-^_pre_)) \| Schmill et al. (2021) \| \|  \|  \| ? \| 3 \| Difference score (CS+ – CS-) for both pretest and posttest \| Chen et al. (2022) \| \|  \|  \|  \|  \| Difference score (CS+ – CS-) posttest only \| Guo et al. (2021) \| \|  \|  \|  \| 2 \| % time spent in CS^+^ \| Marty et al. (2023)^h^ \| \| Cocaine \| Mouse \| ? \| 2 \| Difference score (CS+ – CS-) for both pretest and posttest \| Rulan et al. (2021)^h^ \| \|  \|  \|  \| ?^d^ \| Difference score (CS+ – CS-) for both pretest and posttest \| Zhang K. et al. (2021) \| \| METH \| Rat \| Biased \| 3 \| Difference score (post – pre) \| Yang et al. (2021) \| \|  \|  \|  \|  \| Difference score (CS+ – CS-) for both pretest and posttest \| Liu L. et al. (2022) \| \|  \|  \|  \|  \| Raw time spent in CS^+^ across pretest and posttest \| Yates et al. (2021a); Jiang et al. (2022) \| \|  \|  \|  \| 2 \| Difference score (post – pre) \| Wiah et al. (2023) \| \|  \|  \|  \|  \| Raw time spent in CS^+^ across pretest and posttest \| Li Z. et al. (2021); Reyna et al. (2021) \| \|  \|  \|  \|  \| % time in CS^+^ across pretest and posttest \| Hamdan et al. (2022) \| \|  \|  \|  \| ? \| Raw time spent in CS^+^ across pretest and posttest \| Madden et al. (2021) \| \|  \|  \| Unbiased \| 3 \| Difference score (CS+ – CS-) for both pretest and posttest \| Zhang L. et al. (2021); Chen Y. et al. (2022); Shahveisi et al. (2022a; 2022b); \| \| METH \| Rat \| Unbiased \| 3 \| Difference score (CS+ – CS-) posttest only \| Anooshe et al. (2021); Hassanlou et al. (2021); Khanegheini et al. (2021); Nouri et al. (2021); Majidinezhad et al. (2022); Sharifi et al. (2022); Amirteymori et al. (2023a; 2023b); Nazari-Serenjeh et al. (2023); Veisi et al. (2023); Mirmohammadi et al. (in press) \| \|  \|  \|  \|  \| Ratio calculated as 100*((CS^+^)/(CS^+^ + CS^-^)) \| Danda et al. (2022) \| \|  \|  \| ? \| 3 \| Difference score (CS+ – CS-) for both pretest and posttest \| Yang et al. (2022) \| \|  \| Mouse \| Both^c^ \| 2 \| Difference score (CS+ – CS-) for both pretest and posttest \| Chesworth et al. (2021) \| \|  \|  \| Biased \| 3 \| Difference score (post – pre); Difference score (CS^+^ – CS^-^) for both pretest and posttest \| Busceti et al. (2021) \| \|  \|  \|  \|  \| Difference score (post – pre); Raw time spent CS^+^ across pretest and posttest \| Ru et al. (2022) \| \|  \|  \|  \|  \| Difference score (CS+ – CS-) for both pretest and posttest \| Li Y. et al. (2022a; 2022b); Li J. et al. (2023b) \| \|  \|  \|  \|  \| % Time spent in CS^+^ across pretest and posttest \| Chen H. et al. (2021) \| \| METH \| Mouse \| Biased \| 2 \| Difference score (post – pre) \| Mori et al. (2021); Kusui et al. (2022); Sayson et al. (2022); Su et al. (2022); Potula et al. (2023) \| \|  \|  \|  \|  \| Difference score (CS+ – CS-) for both pretest and posttest \| Qian et al. (2021a; 2021b); Wang Z. et al. (2021); Shang et al. (2022); Tan et al. (2022); Zhu C. et al. (2022); Wang Q. et al. (2023) \| \|  \|  \|  \|  \| Difference score (CS+ – CS-) posttest only \| He et al. (2022) \| \|  \|  \|  \|  \| Raw time spent in CS^+^ across pretest and posttest \| Zhou et al. (2021) \| \|  \|  \| Unbiased \| 3 \| Difference score (CS+ – CS-) for both pretest and posttest \| Cai et al. (2022) \| \|  \|  \|  \|  \| Difference score (CS+ – CS-) posttest only \| Wang Y. et al. (2021) \| \|  \|  \|  \|  \| Raw time spent in CS^+^ across pretest and posttest \| Che et al. (2021); Nukitram et al. (2021) \| \|  \|  \|  \|  \| Ratio calculated as 100*((CS^+^)/(CS^+^ + CS^-^)) \| Cullity et al. (2021) \| \|  \|  \|  \|  \| % of time spent in CS^+^ during posttest relative to time spent in CS^+^ during pretest \| Pham et al. (2022) \| \| METH \| Mouse \| Unbiased \| 2 \| Difference score (CS+ – CS-) posttest only \| Fultz et al. (2021); Shab et al. (2021) \| \|  \|  \|  \|  \| Difference score (post – pre) \| Yan et al. (2022) \| \|  \|  \|  \| ? \| Difference score (post – pre) \| Gu et al. (2021) \| \|  \|  \| ? \| 3 \| Difference score (CS+ – CS-) for both pretest and posttest \| Nukitram et al. (2022a; 2022b; 2023) \| \|  \|  \|  \| 2 \| Raw time spent in CS^+^ across pretest and posttest; Ratio of time spent in CS^+^ during posttest and pretest \| Liu Y. et al. (2022) \| \|  \|  \|  \|  \| Raw time spent in CS^+^ and CS^-^ across pretest and posttest \| Liang et al. (2023) \| \| Nicotine \| Rat \| Biased \| 3 \| Difference score (CS+ – CS-) posttest only \| Salmani et al. (2022) \| \|  \|  \|  \|  \| Difference score (post – pre) presented as % \| Gill et al. (2021) \| \|  \|  \| Unbiased \| 3 \| Difference score (CS+ – CS-) for both pretest and posttest \| Huang et al. (2023) \| \|  \| Mouse \| Biased \| 3 \| Difference score (post – pre) \| Cui et al. (2022) \| \| Nicotine \| Mouse \| Biased \| 3 \| Raw time spent in CS^+^ across pretest and posttest \| Liu G. et al. (2022) \| \|  \|  \|  \|  \| Ratio calculated as 100*(CS^+^_post_/total time) and 100*(CS^-^_post_/total time) and then expressed as % of pretest \| Jia et al. (2021) \| \|  \|  \|  \|  \| % time spent in CS^+^ during posttest compared to % time spent in CS^+^ during pretest (normalized to 100%) \| Nunes-Freitas et al. (2021) \| \|  \|  \|  \| 2 \| Difference score (post – pre) \| Rahmadi et al. (2021) \| \|  \|  \|  \|  \| % difference in CS^+^ across pretest and posttest \| Aranäs et al. (2021) \| \|  \|  \| Unbiased \| 3 \| Difference score (post – pre) \| Akinola et al. (2022); Fan et al. (2023) \| \|  \|  \|  \|  \| Difference score (CS^+^ - CS^-^)_post_ – (CS^+^ - CS^-^)_pre_ \| Cooper et al. (2021); Avelar et al. (2022) \| \|  \|  \|  \|  \| % of time spent in CS^+^ during posttest relative to time spent in CS^+^ during pretest \| D’Souza et al. (2022) \| \|  \|  \|  \| 2 \| Difference score (post – pre) \| de Paula Rosa et al. (2021); Liu et al. (2021) \| \| Nicotine \| Mouse \| Unbiased \| 2 \| Difference score (CS+ – CS-) posttest only \| Noda et al. (2021) \| \|  \|  \|  \|  \| Raw time spent in CS^+^ across pretest and posttest \| Yunusoğlu (2021); Allain et al. (2022) \| \|  \|  \| ? \| 3 \| Ratio calculated as 100*((CS^+^)/(CS^+^ + CS^-^)) \| Luo et al. (2021) \| \|  \|  \|  \|  \|  \|  \| \|  \|  \|  \|  \|  \|  \| \|  \|  \|  \|  \|  \|  \| |
| --- | --- | --- | --- | --- | --- | --- | --- | --- | --- | --- | --- | --- | --- | --- | --- | --- | --- | --- | --- | --- | --- | --- | --- | --- | --- | --- | --- | --- | --- | --- | --- | --- | --- | --- | --- | --- | --- | --- | --- | --- | --- | --- | --- | --- | --- | --- | --- | --- | --- | --- | --- | --- | --- | --- | --- | --- | --- | --- | --- | --- | --- | --- | --- | --- | --- | --- | --- | --- | --- | --- | --- | --- | --- | --- | --- | --- | --- | --- | --- | --- | --- | --- | --- | --- | --- | --- | --- | --- | --- | --- | --- | --- | --- | --- | --- | --- | --- | --- | --- | --- | --- | --- | --- | --- | --- | --- | --- | --- | --- | --- | --- | --- | --- | --- | --- | --- | --- | --- | --- | --- | --- | --- | --- | --- | --- | --- | --- | --- | --- | --- | --- | --- | --- | --- | --- | --- | --- | --- | --- | --- | --- | --- | --- | --- | --- | --- | --- | --- | --- | --- | --- | --- | --- | --- | --- | --- | --- | --- | --- | --- | --- | --- | --- | --- | --- | --- | --- | --- | --- | --- | --- | --- | --- | --- | --- | --- | --- | --- | --- | --- | --- | --- | --- | --- | --- | --- | --- | --- | --- | --- | --- | --- | --- | --- | --- | --- | --- | --- | --- | --- | --- | --- | --- | --- | --- | --- | --- | --- | --- | --- | --- | --- | --- | --- | --- | --- | --- | --- | --- | --- | --- | --- | --- | --- | --- | --- | --- | --- | --- | --- | --- | --- | --- | --- | --- | --- | --- | --- | --- | --- | --- | --- | --- | --- | --- | --- | --- | --- | --- | --- | --- | --- | --- | --- | --- | --- | --- | --- | --- | --- | --- | --- | --- | --- | --- | --- | --- | --- | --- | --- | --- | --- | --- | --- | --- | --- | --- | --- | --- | --- | --- | --- | --- | --- | --- | --- | --- | --- | --- | --- | --- | --- | --- | --- | --- | --- | --- | --- | --- | --- | --- | --- | --- | --- | --- | --- | --- | --- | --- | --- | --- | --- | --- | --- | --- | --- | --- | --- | --- | --- | --- | --- | --- | --- | --- | --- | --- | --- | --- | --- | --- | --- | --- | --- | --- | --- | --- | --- | --- | --- | --- | --- | --- | --- | --- | --- | --- | --- | --- | --- | --- | --- | --- | --- | --- | --- | --- | --- | --- | --- | --- | --- | --- | --- | --- | --- | --- | --- | --- | --- | --- | --- | --- | --- | --- | --- | --- | --- | --- | --- | --- | --- | --- | --- | --- | --- | --- | --- | --- | --- | --- | --- | --- | --- | --- | --- | --- | --- | --- | --- | --- | --- | --- | --- | --- | --- | --- | --- | --- | --- | --- | --- | --- | --- | --- | --- | --- | --- | --- | --- | --- | --- | --- | --- | --- | --- | --- | --- | --- | --- | --- | --- | --- | --- | --- | --- | --- | --- | --- | --- | --- | --- | --- | --- | --- | --- | --- | --- | --- | --- | --- | --- | --- | --- | --- | --- | --- | --- | --- | --- | --- | --- | --- | --- | --- | --- | --- | --- | --- | --- | --- | --- | --- | --- | --- | --- | --- | --- | --- | --- | --- | --- | --- | --- | --- | --- | --- | --- | --- | --- | --- | --- | --- | --- | --- | --- | --- | --- | --- | --- | --- | --- | --- | --- | --- | --- | --- | --- | --- | --- | --- | --- | --- | --- | --- | --- | --- | --- | --- | --- | --- | --- | --- | --- | --- | --- | --- | --- | --- | --- | --- | --- | --- | --- | --- | --- | --- | --- | --- | --- | --- | --- | --- | --- | --- | --- | --- | --- | --- | --- | --- | --- | --- | --- | --- | --- | --- | --- | --- | --- | --- | --- | --- | --- | --- | --- | --- | --- | --- | --- | --- | --- | --- | --- | --- | --- | --- | --- | --- | --- | --- | --- | --- | --- | --- | --- | --- | --- | --- | --- | --- | --- | --- | --- | --- | --- | --- | --- | --- | --- | --- | --- | --- | --- | --- | --- | --- | --- | --- | --- | --- | --- | --- | --- | --- | --- | --- | --- | --- | --- | --- | --- | --- | --- | --- | --- | --- | --- | --- | --- | --- | --- | --- | --- | --- | --- | --- | --- | --- | --- | --- | --- |

Abbreviations: AMPH = amphetamine; METH = methamphetamine

*Notes.* ^a^The type of design was not explicitly stated; however, because the other papers listed with this citation come from the same group, one can infer that a biased design was used in this experiment. ^b^The flooring is interchangeable. During the posttest, flooring that contains both rod flooring and perforated steel flooring. ^c^A biased approach was used for subjects that showed a large preference for one compartment during the pretest. ^d^The CPP apparatus was not described; however, it is implied that a 2-compartment apparatus was used. ^f^How % side preference was calculated was not explicitly detailed. ^g^Examined time spent in both CS^+^ and CS^-^. ^h^Implied that an unbiased design was used, but this was not clearly stated.

**Supplemental References**

Abraham, A. D., Casello, S. M., Land, B. B., and Chavkin, C. (2022). Optogenetic stimulation of dynorphinergic neurons within the dorsal raphe activate kappa opioid receptors in the ventral tegmental area and ablation of dorsal raphe prodynorphin or kappa receptors in dopamine neurons blocks stress potentiation of cocaine reward. *Addict. Neurosci.* 1, 100005. doi: 10.1016/j.addicn.2022.100005

Akinola, L. S., Rahman, Y., Ondo, O., Gonzales, J., Bagdas, D., Jackson A., et al. (2022). Genotypic differences in the effects of menthol on nicotine intake and preference in mice. *Front. Neurosci.* 16, 905330. doi: 10.3389/fnins.2022.905330

Allain, A. E., Aribo, O., Medrano, M. C., Fournier, M. L., Bertrand, S. S., and Caille, S. (2022). Impact of acute and chronic nicotine administration on midbrain dopaminergic neuron activity and related behaviours in TRPV1 knock-out juvenile mice. *Eur. J. Neurosci.* 55, 697-713. doi: 10.1111/ejn.15577

Amaral, I. M., Scheffauer, L., Langeder, A. B., Hofer, A., and El Rawas, R. (2021). Rewarding social interaction in rats increases CaMKII in the nucleus accumbens. *Biomedicines* 9, 1886. doi: 10.3390/biomedicines9121886

Amirteymori, H., Karimi-Haghighi, S., Mirmohammadi, M., Majidinezhad, M., Khosrowabadi, E., and Haghparast, A. (2023a). Hypocretin/orexin system in the nucleus accumbens as a promising player in the extinction and reinstatement of methamphetamine-induced CPP. *Prog. Neuropsychopharmacol. Biol. Psychiatry* 120, 110616. doi: 10.1016/j.pnpbp.2022.110616

Amirteymori, H., Veisi, A., Khaleghzadeh-Ahangar, H., Mozafari, R., and Haghparast, A. (2023b). Involvement of orexin-2 receptors in the CA1 region of the hippocampus in the extinction and reinstatement of methamphetamine-induced conditioned place preference in the rats. *Peptides* 160, 170926. doi: 10.1016/j.peptides.2022.170926

Anooshe, M., Nouri, K., Karimi-Haghighi, S., Mousavi, Z., and Haghparast, A. (2021). Cannabidiol efficiently suppressed the acquisition and expression of methamphetamine-induced conditioned place preference in the rat. *Behav. Brain Res.* 404, 113158. doi: 10.1016/j.bbr.2021.113158

Aranäs, C., Vestlund, J., Witley, S., Edvardsson, C. E., Kalafateli, A. L., and Jerlhag, E. (2021). Salmon calcitonin attenuates some behavioural responses to nicotine in male mice. *Front. Pharmacol.* 12, 685631. doi: 10.3389/fphar.2021.685631

Arenäs, M. C., Castro-Zavala, A., Martín-Sánchez, A., Blanco-Gandía, M. C., Miñarro, J., Valverde, O., et al. (2022). Prepulse inhibition can predict the motivational effects of cocaine in female mice exposed to maternal separation. *Behav. Brain Res.* 416, 113545. doi: 10.1016/j.bbr.2021.113545

Asth, L., Iglesias, L. P., Briânis, R. C., Marçal, A. P., Soares, N. P., Aguiar, D. C., et al. (2021). Effects of the monoamine stabilizer, (-)-OSU6162, on cocaine-induced locomotion and conditioned place preference in mice. *Naunyn Schmiedebergs Arch. Pharmacol.* 394, 1143-1152. doi: 10.1007/s00210-021-02053-x

Atehortua Martinez, L. A., Curis, E., Mekdad, N., Larrieu, C., Courtin, C., et al. (2022). Individual differences in cocaine-induced conditioned place preference in male rats: behavioral and transcriptomic evidence. *J. Psychopharmacol.* 36, 1161-1175. doi: 10.1177/02698811221123047

Avelar, A. J., Cooper, S. Y., Wright, T. D., Wright, S. K., Richardson, M. R., and Henderson, B. J. (2022). Morphine exposure reduces nicotine-induced upregulation of nicotinic receptors and decreases volitional nicotine intake in a mouse model. *Nicotine Tob. Res.* 24, 1161-1168. doi: 10.1093/ntr/ntac002

Babigian, C. J., Wiedner, H. J., Wahlestedt, C., and Sartor, G. C. (2021). JQ1 attenuates psychostimulant- but not opioid-induced conditioned place preference. *Behav. Brain Res.* 418, 113644. doi: 10.1016/j.bbr.2021.113644

Ballestín, R., Alegre-Zurano, L., Ferrer-Pérez, C., Cantacorps, L., Miñarro, J., Valverde, O., et al. (2021). Neuroinflammatory and behavioral susceptibility profile of mice exposed to social stress towards cocaine effects. *Prog. Neuropsychopharmacol. Biol. Psychiatry* 105, 110123. doi: 10.1016/j.pnpbp.2020.110123

Barbosa-Méndez, S., Pérez-Sánchez, G., Becerril-Villanueva, E., and Salazar-Juárez, A. (2021). Melatonin decreases cocaine-induced locomotor sensitization and cocaine-conditioned place preference in rats. *J. Psychiatr. Res.* 132, 97-110. doi: 10.1016/j.jpsychires.2020.09.027

Beiser, T., Lisniansky, E., Weitz, M., Bingor, A., Grad, E., Rosenblum, K., et al. (2022). A functional eEF2K-eEF2 pathway in the NAc is critical for the expression of cocaine-induced psychomotor sensitisation and conditioned place preference. *Transl. Psychiatry* 12, 460. doi: 10.1038/s41398-022-02232-1

Busceti, C. L., Ginerete, R. P., Di Menna, L., D'Errico, G., Cisani, F., Di Pietro, P., et al. (2021). Behavioural and biochemical responses to methamphetamine are differentially regulated by mGlu2 and mGlu3 metabotropic glutamate receptors in male mice. *Neuropharmacology* 196, 108692. doi: 10.1016/j.neuropharm.2021.108692

Caffino, L., Moro, F., Mottarlini, F., Targa, G., Di Clemente, A., Toia, M., et al. (2021). Repeated exposure to cocaine during adolescence enhances the rewarding threshold for cocaine-conditioned place preference in adulthood. *Addict. Biol.* 26, e13012. doi: 10.1111/adb.13012

Cai, J., Che, X., Xu, T., Luo, Y., Yin, M., Lu, X., et al. (2022). Repeated oxytocin treatment during abstinence inhibited context- or restraint stress-induced reinstatement of methamphetamine-conditioned place preference and promoted adult hippocampal neurogenesis in mice. *Exp. Neurol.* 347, 113907. doi: 10.1016/j.expneurol.2021.113907

Calpe-López, C., Gasparyan, A., Navarrete, F., Manzanares, J., Miñarro, J., and Aguilar, M. A. (2021). Cannabidiol prevents priming- and stress-induced reinstatement of the conditioned place preference induced by cocaine in mice. *J. Psychopharmacol.* 35, 864-874. doi: 10.1177/0269881120965952

Calpe-López, C., Martínez-Caballero, M. A., García-Pardo, M. P., and Aguilar, M. A. (2022a). Brief maternal separation inoculates against the effects of social stress on depression-like behavior and cocaine reward in mice. *Front. Pharmacol.* 13, 825522. doi: 10.3389/fphar.2022.825522

Calpe-López, C., Martínez-Caballero, M. A., García-Pardo, M. P., and Aguilar, M. A. (2022b). Intermittent voluntary wheel running promotes resilience to the negative consequences of repeated social defeat in mice. *Physiol. Behav.* 254, 113916. doi: 10.1016/j.physbeh.2022.113916

Chang, H. A., Dai, W., and Hu, S. S. (2021). Sex differences in cocaine-associated memory: the interplay between CB_1_, mGluR5, and estradiol. *Psychoneuroendocrinology* 133, 105366. doi: 10.1016/j.psyneuen.2021.105366

Chang, H. A., Dai, W., and Hu, S. S. Dose-dependent effect of retrieval-extinction on preventing reinstatement of cocaine-associated memory in mice. *Chin. J. Physiol.* 65, 159-170. doi: 10.4103/0304-4920.354804

Che, X., Bai, Y., Cai, J., Liu, Y., Li, Y., Yin, M., et al. (2021). Hippocampal neurogenesis interferes with extinction and reinstatement of methamphetamine-associated reward memory in mice. *Neuropharmacology* 196, 108717. doi: 10.1016/j.neuropharm.2021.108717

Chen, H., Chen, L., Yuan, Z., Yuan, J., Li, Y., Xu, Y., et al. (2022). Glutamate receptor-interacting protein 1 in D1- and D2-dopamine receptor-expressing medium spiny neurons differentially regulates cocaine acquisition, reinstatement, and associated spine plasticity. *Front. Cell Neurosci.* 16, 979078. doi: 10.3389/fncel.2022.979078

Chen, H., Xu, D., Zhang, Y., Yan, Y., Liu, J., Liu, C., et al. (2021). Neurons in the locus coeruleus modulate the hedonic effects of sub-anesthetic dose of propofol. *Front. Neurosci.* 15, 636901. doi: 10.3389/fnins.2021.636901

Chen, Y., Zhang, L., Ding, Z., Wu, X., Wang, G., and Shi, J. (2022). Effects of 3-methylmethcathinone on conditioned place preference and anxiety-like behavior: comparison with methamphetamine. *Front. Mol. Neurosci.* 15, 975820. doi: 10.3389/fnmol.2022.975820

Chesworth, R., Rosa-Porto, R., Yao, S., and Karl, T. (2021). Sex-specific sensitivity to methamphetamine-induced schizophrenia-relevant behaviours in *neuregulin 1 type III* overexpressing mice. *J. Psychopharmacol.* 35, 50-64. doi: 10.1177/0269881120967870

Chou, Y. H., Hor, C. C., Lee, M. T., Lee, H. J., Guerrini, R., Calo, G., et al. (2021). Stress induces reinstatement of extinguished cocaine conditioned place preference by a sequential signaling via neuropeptide S, orexin, and endocannabinoid. *Addict. Biol.* 26, e12971. doi: 10.1111/adb.12971

Christoffel, D. J., Walsh, J. J., Hoerbelt, P., Heifets, B. D., Llorach, P., Lopez, R. C., et al. Selective filtering of excitatory inputs to nucleus accumbens by dopamine and serotonin. (2021). *Proc. Natl. Acad. Sci. U. S. A.* 118, e2106648118. doi: 10.1073/pnas.2106648118

Chu, J., Deyama, S., Li, X., Motono, M., Otoda, A., Saito, A., et al. (2021). Role of 5-HT_1A_ receptor-mediated serotonergic transmission in the medial prefrontal cortex in acute restraint stress-induced augmentation of rewarding memory of cocaine in mice. *Neurosci. Lett.* 743, 135555. doi: 10.1016/j.neulet.2020.135555

Clauss, N. J., Koek, W., and Daws, L. C. (2021). Role of organic cation transporter 3 and plasma membrane monoamine transporter in the rewarding properties and locomotor sensitizing effects of amphetamine in male and female mice. *Int. J. Mol. Sci.* 22, 13420. doi: 10.3390/ijms222413420

Cooper, S. Y., Akers, A. T., Journigan, V. B., and Henderson, B. J. (2021). Novel putative positive modulators of α4β2 nAChRs potentiate nicotine reward-related behavior. *Molecules* 26, 4793. doi: 10.3390/molecules26164793

Cui, J., Ju, X., Lee, Y., Hong, B., Kang, H., Han, K., et al. (2022). Repeated ketamine anesthesia during neurodevelopment upregulates hippocampal activity and enhances drug reward in male mice. *Commun. Biol.* 5, 709. doi: 10.1038/s42003-022-03667-4

Cullity, E. R., Guerin, A. A., Perry, C. J., and Kim, J. H. (2021). Examining sex differences in conditioned place preference or aversion to methamphetamine in adolescent and adult mice. *Front. Pharmacol.* 12, 770614. doi: 10.3389/fphar.2021.770614

Dai, K. Z., Choi, I. B., Levitt, R., Blegen, M. B., Kaplan, A. R., Matsui, A., et al. (2022). Dopamine D2 receptors bidirectionally regulate striatal enkephalin expression: implications for cocaine reward. *Cell Rep.* 40, 111440. doi: 10.1016/j.celrep.2022.111440

Danda, H., Pinterová-Leca, N., Šíchová, K., Štefková-Mazochová, K., Syrová, K., Olejníková, L., et al. (2022). Effects of synthetic cathinone naphyrone in the conditioned place preference test - evidence of its addictive potential. *Behav. Brain Res.* 421, 113713. doi: 10.1016/j.bbr.2021.113713

Darcq, E., Nouel, D., Hernandez, G., Pokinko, M., Ash, P., Moquin, L., et al. (2023). Reduced dopamine release in Dcc haploinsufficiency male mice abolishes the rewarding effects of cocaine but not those of morphine and ethanol. *Psychopharmacology* 240, 637-646. doi: 10.1007/s00213-022-06288-1

da Silva, M. C. M., Gomes, G. F., de Barros Fernandes, H., da Silva, A. M., Teixeira, A. L., Moreira, F. A., et al. (2021). Inhibition of CSF1R, a receptor involved in microglia viability, alters behavioral and molecular changes induced by cocaine. *Sci. Rep.* 11, 15989. doi: 10.1038/s41598-021-95059-7

de Guglielmo, G., Iemolo, A., Nur, A., Turner, A., Montilla-Perez, P., Martinez, A., et al. (2022).Reelin deficiency exacerbates cocaine-induced hyperlocomotion by enhancing neuronal activity in the dorsomedial striatum. *Genes Brain Behav.* 21, e12828. doi: 10.1111/gbb.12828

de Paula Rosa, M. L., Machado, C. A., Oliveira, B. D. S., Toscano, E. C. B., Asth, L., de Barros, J. L. V. M., et al. (2021). Role of cytokine and neurotrophic factors in nicotine addiction in the conditioned place preference paradigm. *Neurosci. Lett.* 764, 136235. doi: 10.1016/j.neulet.2021.136235

D'Souza, M. S., Seeley, S. L., Emerson, N., Rose-Malkamaki, M. J., Ho, S. P., Tsai, Y. C., et al. (2022). Attenuation of nicotine-induced rewarding and antidepressant-like effects in male and female mice lacking regulator of G-protein signaling 2. *Pharmacol. Biochem. Behav.* 213, 173338. doi: 10.1016/j.pbb.2022.173338

Elgueta-Reyes, M., Velásquez, V. B., Espinosa, P., Riquelme, R., Dib, T., Sanguinetti, N. K., et al. (2022). Effects of early life exposure to sex hormones on neurochemical and behavioral responses to psychostimulants in adulthood: implications in drug addiction. *Int. J. Mol. Sci.* 23, 6575. doi: 10.3390/ijms23126575

Fan, L., Chen, H., Liu, Y., Hou, H., and Hu, Q. (2023). ERK signaling is required for nicotine-induced conditional place preference by regulating neuroplasticity genes expression in male mice. *Pharmacol. Biochem. Behav.* 222, 173510. doi: 10.1016/j.pbb.2022.173510

Ferrer-Pérez, C., Reguilón, M. D., Miñarro, J., and Rodríguez-Arias, M. (2022). Effect of voluntary wheel-running exercise on the endocrine and inflammatory response to social stress: conditioned rewarding effects of cocaine. *Biomedicines* 10, 2373. doi: 10.3390/biomedicines10102373

Fontaine, H. M., Silva, P. R., Neiswanger, C., Tran, R., Abraham, A. D., Land, B. B., et al. (2022). Stress decreases serotonin tone in the nucleus accumbens in male mice to promote aversion and potentiate cocaine preference via decreased stimulation of 5-HT_1B_ receptors. *Neuropsychopharmacology* 47, 891-901. doi: 10.1038/s41386-021-01178-0

Fugazzotto, F., Occhiuto, F., García-Pardo, M. P., and Aguilar, M. A. (2021). Hypericum perforatum L. prevents the acquisition of and promotes resilience against stress-induced reinstatement of the conditioned place preference induced by cocaine. *Neurosci. Lett.* 762, 136164. doi: 10.1016/j.neulet.2021.136164

Fultz, E. K., Quadir, S. G., Martin, D., Flaherty, D. M., Worley, P. F., Kippin, T. E., et al. (2021). ERK-directed phosphorylation of mGlu5 gates methamphetamine reward and reinforcement in mouse. *Int. J. Mol. Sci.* 22, 1473. doi: 10.3390/ijms22031473

Galaj, E., Bi, G. H., Moore, A., Chen, K., He, Y., Gardner, E., et al. (2021). Beta-caryophyllene inhibits cocaine addiction-related behavior by activation of PPARα and PPARγ: repurposing a FDA-approved food additive for cocaine use disorder. *Neuropsychopharmacology* 46, 860-870. doi: 10.1038/s41386-020-00885-4

Gárate-Pérez, M. F., Méndez, A., Bahamondes, C., Sanhueza, C., Guzmán, F., Reyes-Parada, M., et al. (2021). Vasopressin in the lateral septum decreases conditioned place preference to amphetamine and nucleus accumbens dopamine release. *Addict. Biol.* 26, e12851. doi: 10.1111/adb.12851

Gill, W. D., Burgess, K. C., Vied, C., and Brown, R. W. (2021). Transgenerational evidence of increases in dopamine D2 receptor sensitivity in rodents: impact on sensorimotor gating, the behavioral response to nicotine and BDNF. *J. Psychopharmacol.* 35, 1188-1203. doi: 10.1177/02698811211033927

Giménez-Gómez, P., Ballestín, R., Gil de Biedma-Elduayen, L., Vidal, R., Ferrer-Pérez, C., Reguilón, M. D., et al. Decreased kynurenine pathway potentiate resilience to social defeat effect on cocaine reward. *Neuropharmacology* 197, 108753. doi: 10.1016/j.neuropharm.2021.108753

Gobira, P. H., Roncalho, A. L., Silva, N. R., Silote, G. P., Sales, A. J., and Joca, S. R. (2021). Adolescent cannabinoid exposure modulates the vulnerability to cocaine-induced conditioned place preference and DNMT3a expression in the prefrontal cortex in Swiss mice. *Psychopharmacology* 238, 3107-3118. doi: 10.1007/s00213-021-05926-4

Gomez, J. L., Bonaventura, J., Keighron, J., Wright, K. M., Marable, D. L., Rodriguez, L. A., et al. (2021). Synaptic Zn^2+^ potentiates the effects of cocaine on striatal dopamine neurotransmission and behavior. *Transl. Psychiatry* 11, 570. doi: 10.1038/s41398-021-01693-0

Gonzalez, A. E., Jorgensen, E. T., Ramos, J. D., Harkness, J. H., Aadland, J. A., Brown, T. E., et al. (2022). Impact of perineuronal net removal in the rat medial prefrontal cortex on parvalbumin interneurons after reinstatement of cocaine conditioned place preference. *Front. Cell Neurosci.* 16, 932391. doi: 10.3389/fncel.2022.932391

Gu, S. M., Seo, S., Park, D., Kim, S., Lamichhane, S., Han, K. M., et al. (2021). Cannabinoid receptor type 1 regulates drug reward behavior via glutamate decarboxylase 67 transcription. *Int. J. Mol. Sci.* 22, 10486. doi: 10.3390/ijms221910486

Guerin, A. A., Zbukvic, I. C., Luikinga, S. J., Drummond, K. D., Lawrence, A. J., Madsen, H. B., et al. (2021). Extinction and drug-induced reinstatement of cocaine seeking following self-administration or conditioned place preference in adolescent and adult rats. *Dev. Psychobiol.* 63, 125-137. doi: 10.1002/dev.22017

Guerrero-Bautista, R., Franco-García, A., Hidalgo, J. M., Fernández-Gómez, F. J., Ribeiro Do Couto, B., Milanés, M. V., et al. (2021). Distinct regulation of dopamine D3 receptor in the basolateral amygdala and dentate gyrus during the reinstatement of cocaine CPP induced by drug priming and social stress. *Int. J. Mol. Sci.* 22, 3100. doi: 10.3390/ijms22063100

Guo, M. L., Chivero, E. T., Callen, S. E., and Buch, S. (2021). NLRP3 Inflammasome is involved in cocaine-mediated potentiation on behavioral changes in CX3CR1-deficient mice. *J. Pers. Med.* 11, 963. doi: 10.3390/jpm11100963

Guzman, A. S., Avalos, M. P., De Giovanni, L. N., Euliarte, P. V., Sanchez, M. A., Mongi-Bragato, B., et al. (2021). CB1R activation in nucleus accumbens core promotes stress-induced reinstatement of cocaine seeking by elevating extracellular glutamate in a drug-paired context. *Sci. Rep.* 11, 12964. doi: 10.1038/s41598-021-92389-4

Hamdan, J. N., Sierra-Fonseca, J. A., Flores, R. J., Saucedo, S. Jr, Miranda-Arango, M., O'Dell, L. E., et al. (2022). Early-life adversity increases anxiety-like behavior and modifies synaptic protein expression in a region-specific manner. *Front. Behav. Neurosci.* 16, 1008556. doi: 10.3389/fnbeh.2022.1008556

Hammad, A. M., Alasmari, F., and Sari, Y. (2021). Effect of modulation of the astrocytic glutamate transporters' expression on cocaine-induced reinstatement in male P rats exposed to ethanol. *Alcohol Alcohol.* 56, 210-219. doi: 10.1093/alcalc/agaa104

Harraz, M. M., Guha, P., Kang, I. G., Semenza, E. R., Malla, A. P., Song, Y. J., et al. (2021). Cocaine-induced locomotor stimulation involves autophagic degradation of the dopamine transporter. *Mol. Psychiatry* 26, 370-382. doi: 10.1038/s41380-020-00978-y

Hassanlou, A. A., Jamali, S., RayatSanati, K., Mousavi, Z., and Haghparast, A. (2021). Cannabidiol modulates the METH-induced conditioned place preference through D2-like dopamine receptors in the hippocampal CA1 region. *Brain Res. Bull.* 172, 43-51. doi: 10.1016/j.brainresbull.2021.04.007

He, T., Chen, W., Fan, Y., Xu, X., Guo, H., Li, N., et al. (2022). A novel cholinergic projection from the lateral parabrachial nucleus and its role in methamphetamine-primed conditioned place preference. *Brain Commun.* 4, fcac219. doi: 10.1093/braincomms/fcac219

Hillhouse, T. M., Olson, K. M., Hallahan, J. E., Rysztak, L. G., Sears, B. F., Meurice, C., et al. The buprenorphine analogue BU10119 attenuates drug-primed and stress-induced cocaine reinstatement in mice. *J. Pharmacol. Exp. Ther.* 378, 287-299. doi: 10.1124/jpet.121.000524

Hilz, E. N., Olvera, M. E., Jun, D., Chadha, M., Gillette, R., Monfils, M. H., et al. (2022). Hormonal contraceptives alter amphetamine place preference and responsivity in the intact female rat. *Behav. Neurosci.* 136, 318-329. doi: 10.1037/bne0000520

Huang, E. Y., Hung, H. Y., Chen, Y. H., Kao, J. H., Tsai, A. L., and Chow, L. H. (2023). Effects of dextromethorphan on nicotine-induced reward, behavioral sensitization, withdrawal signs, and drug seeking-related behavior in rats. *Nicotine Tob. Res.* 25, 1251-1260. doi: 10.1093/ntr/ntac287

Inbar, K., Levi, L. A., and Kupchik, Y. M. (2022). Cocaine induces input and cell-type-specific synaptic plasticity in ventral pallidum-projecting nucleus accumbens medium spiny neurons. *Neuropsychopharmacology* 47, 1461-1472. doi: 10.1038/s41386-022-01285-6

Jia, W., Wilar, G., Kawahata, I., Cheng, A., and Fukunaga, K. (2021). Impaired acquisition of nicotine-induced conditioned place preference in fatty acid-binding protein 3 null mice. *Mol. Neurobiol.* 58, 2030-2045. doi: 10.1007/s12035-020-02228-2

Jiang, S. Z., Sweat, S., Dahlke, S. P., Loane, K., Drossel, G., Xu, W., et al. (2021). Cocaine-dependent acquisition of locomotor sensitization and conditioned place preference requires D1 dopaminergic signaling through a cyclic AMP, NCS-rapgef2, ERK, and Egr-1/Zif268 pathway. *J. Neurosci.* 41, 711-725. doi: 10.1523/JNEUROSCI.1497-20.2020

Jiang, C., Xu, Y., Zhong, J., Wu, J., He, J., Xu, W., et al. (2022). Chloral hydrate alters brain activation induced by methamphetamine-associated cue and prevents relapse. *Front. Mol. Neurosci.* 15, 934167. doi: 10.3389/fnmol.2022.934167

Jorgensen, E. T., Gonzalez, A. E., Harkness, J. H., Hegarty, D. M., Thakar, A., Burchi, D. J., et al. Cocaine memory reactivation induces functional adaptations within parvalbumin interneurons in the rat medial prefrontal cortex. *Addict. Biol.* 26, e12947. doi: 10.1111/adb.12947

Kähler, B., Romswinkel, E. V., Jakovcevski, M., Moses, A., Schachner, M., and Morellini, F. (2021). Hyperfunction of the stress response system and novelty-induced hyperactivity correlate with enhanced cocaine-induced conditioned place preference in NCAM-deficient mice. *Addict. Biol.* 26, e12887. doi: 10.1111/adb.12887

Kalafateli, A. L., Aranäs, C., and Jerlhag, E. (2021). Activation of the amylin pathway modulates cocaine-induced activation of the mesolimbic dopamine system in male mice. *Horm. Behav.* 127, 104885. doi: 10.1016/j.yhbeh.2020.104885

Kawahara, Y., Ohnishi, Y. N., Ohnishi, Y. H., Kawahara, H., and Nishi, A. (2021). Distinct role of dopamine in the PFC and NAc during exposure to cocaine-associated cues. *Int. J. Neuropsychopharmacol.* 24, 988-1001. doi: 10.1093/ijnp/pyab067

Khanegheini, A., Khani, M., Zarrabian, S., Yousefzadeh-Chabok, S., Taleghani, B. K., and Haghparast, A. (2021). Cannabidiol enhanced the development of sensitization to the expression of methamphetamine-induced conditioned place preference in male rats. *J. Psychiatr. Res.* 137, 260-265. doi: 10.1016/j.jpsychires.2021.02.045

Kołosowska, K., Lehner, M., Skórzewska, A., Gawryluk, A., Tomczuk, F., Sobolewska, A., et al. (2023). Molecular pattern of a decrease in the rewarding effect of cocaine after an escalating-dose drug regimen. *Pharmacol. Rep.* 75, 85-98. doi: 10.1007/s43440-022-00443-3

Kusui, Y., Izuo, N., Uno, K., Ge, B., Muramatsu, S. I., and Nitta, A. (2022). Knockdown of piccolo in the nucleus accumbens suppresses methamphetamine-induced hyperlocomotion and conditioned place preference in mice. *Neurochem. Res.* 47, 2856-2864. doi: 10.1007/s11064-022-03680-3

Ledesma, J. C., Manzanedo, C., and Aguilar, M. A. (2021). Cannabidiol prevents several of the behavioral alterations related to cocaine addiction in mice. *Prog. Neuropsychopharmacol. Biol. Psychiatry* 111, 110390. doi: 10.1016/j.pnpbp.2021.110390

Lemos, C., Salti, A., Amaral, I. M., Fontebasso, V., Singewald, N., Dechant, G., et al. (2021). Social interaction reward in rats has anti-stress effects. *Addict. Biol.* 26, e12878. doi: 10.1111/adb.12878

Li, Z., Qi, Y., Liu, K., Cao, Y., Zhang, H., Song, C., et al. (2021). Effect of Chaihu-jia-Longgu-Muli decoction on withdrawal symptoms in rats with methamphetamine-induced conditioned place preference. *Biosci. Rep.* 41, BSR20211376. doi: 10.1042/BSR20211376

Li, H., Wan, X., Wu, Z., Zhou, Y., Chen, R., Xu, W., et al. (2022). β-hydroxybutyrate reduces reinstatement of cocaine conditioned place preference through hippocampal CaMKII-α β-hydroxybutyrylation. *Cell Rep.* 41, 111724. doi: 10.1016/j.celrep.2022.111724

Li, Y., Re, G. F., Zhao, Y., Kong, D., Mao, J. H., Wang, K. H., et al. (2022a). Messenger RNA expression profiles and bioinformatics analysis of mouse hippocampi during exercise alleviates methamphetamine dependence via mRNA profile change in hippocampi. *Ann. Transl. Med.* 10, 957. doi: 10.21037/atm-22-450

Li, Y., Re, G. F., Zhao, Y., Wu, X. C., Zhou, R. Y., Kuang, Y.Q., et al. (2022b). Long-term exercise at different intensities can reduce the inflammatory response in the brains of methamphetamine-treated mice. *Biochem. Biophys. Res. Commun.* 613, 201-206. doi: 10.1016/j.bbrc.2022.05.042

Li, W., Zhang, C., Wang, Y. Y., Xiao, L., Feng, Y., Huo, X., et al. (2022). Alterations of RNAs in the insula related to cocaine-induced condition place preference in adolescent mice. *Biochem. Biophys. Res. Commun.* 621, 109-115. doi: 10.1016/j.bbrc.2022.06.080

Li, J., Wu, Y., Xue, T., He, J., Zhang, L., Liu, Y., et al. (2023a). Cdc42 signaling regulated by dopamine D2 receptor correlatively links specific brain regions of hippocampus to cocaine addiction. *Biochim. Biophys. Acta Mol. Basis Dis.* 1869, 166569. doi: 10.1016/j.bbadis.2022.166569

Li, J. Y., Yu, Y. J., Su, C. L., Shen, Y. Q., Chang, C. H., and Gean, P. W. (2023b). Modulation of methamphetamine memory reconsolidation by neural projection from basolateral amygdala to nucleus accumbens. *Neuropsychopharmacology* 48, 478-488. doi: 10.1038/s41386-022-01417-y

Liang, M., Chen, G., Xi, Z., Qian, H., Shang, Q., Gao, B., et al. (2023). The roles of K^+^-dependent Na^+^/Ca^2+^ exchanger 2 (NCKX2) in methamphetamine-induced behavioral sensitization and conditioned place preference in mice. *Neurosci. Lett.* 792, 136952. doi: 10.1016/j.neulet.2022.136952

Liu, G., Wang, R., Chen, H., Wu, P., Fu, Y., Li, K., et al. (2022b). Non-nicotine constituents in cigarette smoke extract enhance nicotine addiction through monoamine oxidase A inhibition. *Front. Neurosci.* 16, 1058254. doi: 10.3389/fnins.2022.1058254

Liu, L., Li, J., Wang, C., Xu, Y., Leung, C. K., Yang, G., et al. (2022a). Cannabidiol attenuates methamphetamine-induced conditioned place preference in male rats and viability in PC12 cells through the Sigma1R/AKT/GSK3β/CREB signaling pathway. *Am. J. Drug Alcohol Abuse* 48, 548-561. doi: 10.1080/00952990.2022.2073450

Liu, Y., Wu, M., Sun, Z., Li, Q., Jiang, R., Meng, F., et al. (2022c). Effect of PPM1F in dorsal raphe 5-HT neurons in regulating methamphetamine-induced conditioned place preference performance in mice. *Brain Res. Bull.* 179, 36-48. doi: 10.1016/j.brainresbull.2021.12.001

Liu, Q., Yu, J., Li, X., Guo, Y., Sun, T., Luo, L., et al. (2021). Cannabinoid receptor GPR55 activation blocks nicotine use disorder by regulation of AMPAR phosphorylation. *Psychopharmacology* 238, 3335-3346. doi: 10.1007/s00213-021-05949-x

Luo, J., Bian, L. H., Yao, Z. W., Wang, X. M., Li, Q. Y., Guo, J. Y., et al. (2021). Anthocyanins in Lycium ruthenicum Murray reduce nicotine withdrawal-induced anxiety and craving in mice. *Neurosci. Lett.* 763, 136152. doi: 10.1016/j.neulet.2021.136152

Madden, J. T., Reyna, N. C., Goranson, E. V., Gonzalez, T. A., Zavala, A. R., and Pentkowski, N. S. (2021). Blocking serotonin 2A (5-HT_2A_) receptors attenuates the acquisition of methamphetamine-induced conditioned place preference in adult female rats. *Behav. Brain Res.* 415, 113521. doi: 10.1016/j.bbr.2021.113521

Majidinezhad, M., Amirteymouri, H., Karimi-Haghighi, S., Kourosh-Arami, M., and Haghparast, A. (2022). Orexin system in the ventral tegmental area is implicated in the rewarding properties of methamphetamine. *Eur. J. Pharmacol.* 930, 175170. doi: 10.1016/j.ejphar.2022.175170

Mañas-Padilla, M. C., Gil-Rodríguez, S., Sampedro-Piquero, P., Ávila-Gámiz, F., Rodríguez de Fonseca, F., Santín, L. J., et al. (2021). Remote memory of drug experiences coexists with cognitive decline and abnormal adult neurogenesis in an animal model of cocaine-altered cognition. *Addict. Biol.* 26, e12886. doi: 10.1111/adb.12886

Mannangatti, P., Ragu Varman, D., Ramamoorthy, S., and Jayanthi, L. D. (2021). Neurokinin-1 antagonism distinguishes the role of norepinephrine transporter from dopamine transporter in mediating amphetamine behaviors. *Pharmacology* 106, 597-605. doi: 10.1159/000518033

Martínez-Rivera, A., Hao, J., Rice, R., Inturrisi, C. E., Rajadhyaksha, A. M. (2023). Ca_v_1.3 L-type Ca^2+^ channel-activated CaMKII/ERK2 pathway in the ventral tegmental area is required for cocaine conditioned place preference. *Neuropharmacology* 224, 109368. doi: 10.1016/j.neuropharm.2022.109368

Marty, V., Butler, J. J., Coutens, B., Chargui, O., Chagraoui, A., Guiard, B. P., et al. (2023). Deleting Snord115 genes in mice remodels monoaminergic systems activity in the brain toward cortico-subcortical imbalances. *Hum. Mol. Genet.* 32, 244-261. doi: 10.1093/hmg/ddac139

Melchior, J. R., Perez, R. E., Salimando, G. J., Luchsinger, J. R., Basu, A., and Winder, D. G. (2021). Cocaine augments dopamine mediated inhibition of neuronal activity in the dorsal bed nucleus of the stria terminalis. *J. Neurosci.* 41, 5876–5893. doi: 10.1523/JNEUROSCI.0284-21.2021

Meng, S., Yan, W., Liu, X., Gong, Y., Tian, S., Wu, P., et al. (2021). Social interaction with relapsed partner facilitates cocaine relapse in rats. *Front. Pharmacol.* 12, 750397. doi: 10.3389/fphar.2021.750397

Metz, V. G., da Rosa, J. L. O., Rossato, D. R., Burger, M. E., and Pase, C. S. (2022). Cannabidiol treatment prevents drug reinstatement and the molecular alterations evoked by amphetamine on receptors and enzymes from dopaminergic and endocannabinoid systems in rats. *Pharmacol. Biochem. Behav.* 218, 173427. doi: 10.1016/j.pbb.2022.173427

Metz, V. G., da Rosa, J. L. O., Rossato, D. R., Milanesi, L. H., Burger, M. E., and Pase, C. S. (2021). Cannabidiol prevents amphetamine relapse and modulates D1- and D2-receptor levels in mesocorticolimbic brain areas of rats. *Eur. Neuropsychopharmacol.* 50, 23-33. doi: 10.1016/j.euroneuro.2021.04.008

Migliaro, M., Sánchez-Zavaleta, R., Soto-Tinoco, E., Ruiz-Contreras, A. E., Méndez-Díaz, M., Herrera-Solís, A., et al. (2022). Dominance status is associated with a variation in cannabinoid receptor 1 expression and amphetamine reward. *Pharmacol. Biochem. Behav.* 221, 173483. doi: 10.1016/j.pbb.2022.173483

Mirmohammadi, M., Eskandari, K., Koruji, M., Shabani, R., Ahadi, R., and Haghparast, A. (in press). Intra-accumbal D1- but not D2-like dopamine receptor antagonism reverses the inhibitory effects of cannabidiol on extinction and reinstatement of methamphetamine seeking behavior in rats. *Cannabis Cannabinoid Res.* doi: 10.1089/can.2022.0017

Montagud-Romero, S., Reguilón, M. D., Pascual, M., Blanco-Gandía, M. C., Guerri, C., Miñarro, J., and Rodríguez-Arias, M. (2021). Critical role of TLR4 in uncovering the increased rewarding effects of cocaine and ethanol induced by social defeat in male mice. *Neuropharmacology* 182, 108368. doi: 10.1016/j.neuropharm.2020.108368

Mori, T., Uzawa, N., Masukawa, D., Hirayama, S., Iwase, Y., Hokazono, M., et al. (2021). Enhancement of the rewarding effects of 3,4-methylenedioxymethamphetamine in orexin knockout mice. *Behav. Brain Res.* 396, 112802. doi: 10.1016/j.bbr.2020.112802

Mukherjee, D., Gonzales, B. J., Ashwal-Fluss, R., Turm, H., Groysman, M., and Citri, A. (2021). Egr2 induction in spiny projection neurons of the ventrolateral striatum contributes to cocaine place preference in mice. *Elife* 10, e65228. doi: 10.7554/eLife.65228

Nazari-Serenjeh, F., Azizbeigi, R., Rashvand, M., Mesgar, S., Amirteymori, H., and Haghparast, A. (2023). Distinct roles for orexin-1 and orexin-2 receptors in the dentate gyrus of the hippocampus in the methamphetamine-seeking behavior in the rats. *Life Sci.* 312, 121262. doi: 10.1016/j.lfs.2022.121262

Nedelescu, H., Wagner, G. E., De Ness, G. L., Carroll, A., Kerr, T. M., Wang, J., et al. (2022). Cannabidiol produces distinct u-shaped dose-response effects on cocaine-induced conditioned place preference and associated recruitment of prelimbic neurons in male rats. *Biol. Psychiatry Glob. Open Sci.* 2, 70-78. doi: 10.1016/j.bpsgos.2021.06.014

Nouri, K., Anooshe, M., Karimi-Haghighi, S., Mousavi, Z., and Haghparast, A. (2021). Involvement of hippocampal d1-like dopamine receptors in the inhibitory effect of cannabidiol on acquisition and expression of methamphetamine-induced conditioned place preference. *Neurochem. Res.* 46, 2008-2018. doi: 10.1007/s11064-021-03350-w

Nguyen, A. T. M., Quach, T. V. B., Kotha, P., Chien, S. Y., MacDonald, I. J., Lane, H. Y., et al. (2021). Electroacupuncture prevents cocaine-induced conditioned place preference reinstatement and attenuates ΔFosB and GluR2 expression. *Sci. Rep.* 11, 13694. doi: 10.1038/s41598-021-93014-0

Noda, Y., Soeda, K., Uchida, M., Goto, S., Ito, T., Kitagaki, S., et al. (2021). Multiple nicotinic acetylcholine receptor subtypes regulate social or cognitive behaviors in mice repeatedly administered phencyclidine. *Behav. Brain Res.* 408, 113284. doi: 10.1016/j.bbr.2021.113284

Nukitram, J., Cheaha, D., and Kumarnsit, E. (2021). Spectral power and theta-gamma coupling in the basolateral amygdala related with methamphetamine conditioned place preference in mice. *Neurosci. Lett.* 756, 135939. doi: 10.1016/j.neulet.2021.135939

Nukitram, J., Cheaha, D., Sengnon, N., Wungsintaweekul, J., Limsuwanchote, S., and Kumarnsit, E. (2022a). Ameliorative effects of alkaloid extract from Mitragyna speciosa (Korth.) Havil. leaves on methamphetamine conditioned place preference in mice. *J. Ethnopharmacol.* 284, 114824. doi: 10.1016/j.jep.2021.114824

Nukitram, J., Cheaha, D., Thawaii, S., Niyomdecha, S., and Kumarnsit, E. (2022b). Neural signaling of methamphetamine craving and seeking intensified by bupropion in the ventral tegmental area-cortico-accumbens circuitry in mice. *Addict. Biol.* 27, e13240. doi: 10.1111/adb.13240

Nukitram, J., Kumarnsit, E., and Cheaha, D. (2023). A 1:1 ratio of cannabidiol: tetrahydrocannabinol attenuates methamphetamine conditioned place preference in mice: a prospective study of antidopaminergic mechanism. *Brain Res. Bull.* 192, 47-55. doi: 10.1016/j.brainresbull.2022.11.003

Nunes-Freitas, A. L., Manhães, A. C., Dutra-Tavares, A. C., Leal-Rocha, P. H., Filgueiras, C. C., Ribeiro-Carvalho, A., et al. (2021). Sex- and age-dependent differences in nicotine susceptibility evoked by developmental exposure to tobacco smoke and/or ethanol in mice. *J. Dev. Orig. Health Dis.* 12, 940-951. doi: 10.1017/S2040174420001191

Panopoulou, M., and Schlüter, O. M. (2022). Ca^2+^-permeable AMPA receptors set the threshold for retrieval of drug memories. *Mol. Psychiatry* 27, 2868-2878. doi: 10.1038/s41380-022-01505-x

Parrilla-Carrero, J., Eid, M., Li, H., Chao, Y. S., and Jhou, T. C. (2021). Synaptic adaptations at the rostromedial tegmental nucleus underlie individual differences in cocaine avoidance behavior. *J. Neurosci.* 41, 4620-4630. doi: 10.1523/JNEUROSCI.1847-20.2021

Pastor, V., Castillo Díaz, F., Sanabria, V. C., Dalto, J. F., Antonelli, M. C., and Medina, J. H. (2021). Prefrontal cortex nicotinic receptor inhibition by methyllycaconitine impaired cocaine-associated memory acquisition and retrieval. *Behav. Brain Res.* 406, 113212. doi: 10.1016/j.bbr.2021.113212

Pham, H., Sieg, J., Seeley, S. L., and D'Souza, M. S. (2022). Differential methamphetamine-induced behavioral effects in male and female mice lacking regulator of G Protein signaling 4. *Behav. Brain Res.* 423, 113770. doi: 10.1016/j.bbr.2022.113770

Philogene-Khalid, H. L., Morrison, M. F., Darbinian, N., Selzer, M. E., Schroeder, J., and Rawls, S. M. (2022). The GLT-1 enhancer clavulanic acid suppresses cocaine place preference behavior and reduces GCPII activity and protein levels in the rat nucleus accumbens. *Drug Alcohol Depend.* 232, 109306. doi: 10.1016/j.drugalcdep.2022.109306

Potula, R., Gentile, T. A., Meissler, J. J., Shekarabi, A., Wiah, S., Farkas, D. J., et al. (2023). Purinergic P2X7 receptor antagonist inhibits methamphetamine-induced reward, hyperlocomotion, and cortical IL-7A levels in mice: a role for P2X7/IL-17A crosstalk in methamphetamine behaviors? *Brain Behav. Immun.* 107, 47-52. doi: 10.1016/j.bbi.2022.09.012

Preston, C. J., and Wagner, J. J. (2022). Withdrawal from cocaine conditioning progressively alters AMPA receptor-mediated transmission in the ventral hippocampus. *Addict. Biol.* 27, e13101. doi: 10.1111/adb.13101

Pujante-Gil, S., Manzanedo, C., and Arenas, M. C. (2021). Sex differences in behavioral traits related with high sensitivity to the reinforcing effects of cocaine. *Behav. Brain Res.* 414, 113505. doi: 10.1016/j.bbr.2021.113505

Qian, H., Shang, Q., Liang, M., Gao, B., Xiao, J., Wang, J., et al. (2021a). MicroRNA-31-3p/RhoA signaling in the dorsal hippocampus modulates methamphetamine-induced conditioned place preference in mice. *Psychopharmacology* 238, 3207-3219. doi: 10.1007/s00213-021-05936-2

Qian, H., Xiao, J., Shang, Q., Wang, J., Liang, M., Gao, B., et al. (2021b). Spatiotemporal expression of Rap1 and Ras mediates the acquisition and reinstatement of methamphetamine-induced conditioned place preference in mice via extracellular signal-regulated kinase activation. *Neuroreport* 32, 1035-1040. doi: 10.1097/WNR.0000000000001686

Quigley, J. A., and Becker, J. B. (2021). Activation of G-protein coupled estradiol receptor 1 in the dorsolateral striatum attenuates preference for cocaine and saccharin in male but not female rats. *Horm. Behav.* 130, 104949. doi: 10.1016/j.yhbeh.2021.104949

Ragu Varman, D., Mannangatti, P., Subler, M. A., Windle, J. J., Ramamoorthy, S., and Jayanthi, L. D. (2022). Blunted amphetamine-induced reinforcing behaviors and transporter downregulation in knock-in mice carrying alanine mutations at threonine-258 and serine-259 of norepinephrine transporter. *J. Mol. Neurosci.* 72, 1965-1976. doi: 10.1007/s12031-022-01988-x

Rahmadi, M., Suasana, D., Lailis, S. R., Ratri, D. M. N., and Ardianto, C. (2021). The effects of quercetin on nicotine-induced reward effects in mice. *J. Basic Clin. Physiol. Pharmacol.* 32, 327-333. doi: 10.1515/jbcpp-2020-0418

Reguilón, M. D., Ballestín, R., Miñarro, J., and Rodríguez-Arias, M. (2022). Resilience to social defeat stress in adolescent male mice. *Prog. Neuropsychopharmacol. Biol. Psychiatry* 119, 110591. doi: 10.1016/j.pnpbp.2022.110591

Reyna, N. C., Madden, J. T., Thiel, K. J., and Pentkowski, N. S. (2021). Methamphetamine and social rewards interact to produce enhanced conditioned place preference in male adolescent rats. *Pharmacol. Biochem. Behav.* 201, 173091. doi: 10.1016/j.pbb.2020.173091

Ródenas-González, F., Blanco-Gandía, M. D. C., Miñarro López, J., and Rodriguez-Arias, M. (2021a). Behavioral and neuroimmune characterization of resilience to social stress: rewarding effects of cocaine. *Adicciones.* 33, 319-332. doi: 10.20882/adicciones.1348

Ródenas-González, F., Blanco-Gandía, M. D. C., Pascual, M., Molari, I., Guerri, C., López, J. M., et al. (2021b). A limited and intermittent access to a high-fat diet modulates the effects of cocaine-induced reinstatement in the conditioned place preference in male and female mice. *Psychopharmacology* 238, 2091-2103. doi: 10.1007/s00213-021-05834-7

Ródenas-González, F., Blanco-Gandía, M. C., Miñarro, J., and Rodríguez-Arias, M. (2022). Effects of ketosis on cocaine-induced reinstatement in male mice. *Neurosci. Lett.* 778, 136619. doi: 10.1016/j.neulet.2022.136619

Rohan, M. L., Lowen, S. B., Rock, A., and Andersen, S. L. (2021). Novelty preferences and cocaine-associated cues influence regions associated with the salience network in juvenile female rats. *Pharmacol. Biochem. Behav.* 203, 173117. doi: 10.1016/j.pbb.2021.173117

Rosa, M. L. P., Machado, C. A., Asth, L., Toscano, E. C. B., da Silva Oliveira, B., Marzano, L. A. S., et al. (2022). A three-compartment apparatus alters the brain concentration of cytokines and neurotrophic factors in cocaine-induced CPP in mice. *J. Neuroimmunol.* 369, 577914. doi: 10.1016/j.jneuroim.2022.577914

Rossato, D. R., Rosa, H. Z., Rosa, J. L. O., Milanesi, L. H., Metz, V. G., D'Àvila, L. F., et al. (2022). Tactile stimulation in adult rats modulates dopaminergic molecular parameters in the nucleus accumbens preventing amphetamine relapse. *Mol. Neurobiol.* 59, 5564-5573. doi: 10.1007/s12035-022-02927-y

Ru, Q., Xiong, Q., Tian, X., Xu, C., Li, C., Chen, L., et al. (2022). Candidate Chinese herbal medicine alleviates methamphetamine addiction *via* regulating dopaminergic and serotonergic pathways. *Front. Mol. Neurosci.* 15, 874080. doi: 10.3389/fnmol.2022.874080

Rulan, D., Zhenbang, Y., Yipu, Z., Yuan, G., Galaj, E., Xiaorui, S., et al. (2021). Exogenous SO_2_ donor treatment impairs reconsolidation of drug reward memory in mice. *Eur. J. Pharmacol.* 896, 173911. doi: 10.1016/j.ejphar.2021.173911

Salmani, N., Nozari, M., Parvan, M., Amini-Sardouei, S., Shabani, M., Khaksari, M., et al. (2022). Nicotine-conditioned place preference, reversal learning and social interaction in MK-801-induced schizophrenia model: effects of post-weaning enriched environment. *Clin. Exp. Pharmacol. Physiol.* 49, 871-880. doi: 10.1111/1440-1681.13674

Sánchez-Zavaleta, R., Segovia, J., Ruiz-Contrerasm, A. E., Herrera-Solís, A., Méndez-Díaz, M., de la Mora, M. P., et al. (2023). GPR55 activation prevents amphetamine-induced conditioned place preference and decrease the amphetamine-stimulated inflammatory response in the ventral hippocampus in male rats. *Prog. Neuropsychopharmacol. Biol. Psychiatry* 120, 110636. doi: 10.1016/j.pnpbp.2022.110636

Sayson, L. V., Kim, M., Jeon, S. J., Custodio, R. J. P., Lee, H. J., Ortiz, D. M., et al. (2022). Differentially expressed genes in *period 2*-overexpressing mice striatum may underlie their lower sensitivity to methamphetamine addiction-like behavior. *Biomol. Ther.* 30, 238-245. doi: 10.4062/biomolther.2021.184

Schmill, M. P., Cadney, M. D., Thompson, Z., Hiramatsu, L., Albuquerque, R. L., McNamara, M. P., et al. (2021). Conditioned place preference for cocaine and methylphenidate in female mice from lines selectively bred for high voluntary wheel-running behavior. *Genes Brain Behav.* 20, e12700. doi: 10.1111/gbb.12700

Segat, H. J., Martini, F., Roversi, K., Rosa, S. G., Muller, S. G., Rossato, D. R., et al. (2022). Impact of two different types of exercise training on AMPH addiction: role of hippocampal neurotrophins. *Physiol. Behav.* 251, 113804. doi: 10.1016/j.physbeh.2022.113804

Shab, G., Fultz, E. K., Page, A., Coelho, M. A., Brewin, L. W., Stailey, N., et al. (2021). The motivational valence of methamphetamine relates inversely to subsequent methamphetamine self-administration in female C57BL/6J mice. *Behav. Brain Res.* 398, 112959. doi: 10.1016/j.bbr.2020.112959

Shahen-Zoabi, S., Smoum, R., Beiser, T., Nemirovski, A., Mechoulam, R., and Yaka, R. (in press). N-oleoyl glycine and its derivatives attenuate the acquisition and expression of cocaine-induced behaviors. *Cannabis Cannabinoid Res.* doi: 10.1089/can.2022.0005

Shahveisi, K., Abdoli, N., Farnia, V., Khazaie, H., Hosseini, M., Ghazvini, H., et al. (2022a). REM sleep deprivation before extinction or reinstatement alters methamphetamine reward memory via D1-like dopamine receptors. *Pharmacol. Biochem. Behav.* 213, 173319. doi: 10.1016/j.pbb.2021.173319

Shahveisi, K., Abdoli, N., Khazaie, H., Farnia, V., and Khodamoradi, M. (2022b). Maternal sleep deprivation affects extinction and reinstatement of methamphetamine reward memory in male offspring: role of the D1-like and D2-like dopamine receptors. *Brain Res.* 1792, 148033. doi: 10.1016/j.brainres.2022.148033

Shang, Q., Wang, J., Xi, Z., Gao, B., Qian, H., An, R., et al. (2022). Mechanisms underlying microRNA-222-3p modulation of methamphetamine-induced conditioned place preference in the nucleus accumbens in mice. *Psychopharmacology* 239, 2997-3008. doi: 10.1007/s00213-022-06183-9

Sharifi, A., Karimi-Haghighi, S., Shabani, R., Asgari, H. R., Ahadi, R., and Haghparast, A. (2022). Cannabidiol impairs the rewarding effects of methamphetamine: Involvement of dopaminergic receptors in the nucleus accumbens. *Prog. Neuropsychopharmacol. Biol. Psychiatry* 113, 110458. doi: 10.1016/j.pnpbp.2021.110458

Shaw, J. K., Pamela Alonso, I., Lewandowski, S. I., Scott, M. O., O'Connor, B. M., Aggarwal, S., et al. (2021). Individual differences in dopamine uptake in the dorsomedial striatum prior to cocaine exposure predict motivation for cocaine in male rats. *Neuropsychopharmacology* 46, 1757-1767. doi: 10.1038/s41386-021-01009-2

Shetty, R. A., Rutledge, M., LeBouf, A., Mock, J. T., Pathak, G., and Forster, M. J. (2022). Expression of stable and reliable preference and aversion phenotypes following place conditioning with psychostimulants. *Psychopharmacology* 239, 2593-2603. doi: 10.1007/s00213-022-06130-8

Shi, X., von Weltin, E., Fitzsimmons, E., Do, C., Caban Rivera, C., Chen, C., et al. (2022). Reactivation of cocaine contextual memory engages mechanistic target of rapamycin/S6 kinase 1 signaling. *Front. Pharmacol.* 13, 976932. doi: 10.3389/fphar.2022.976932

Siemian, J. N., Arenivar, M. A., Sarsfield, S., Borja, C. B., Russell, C. N., and Aponte, Y. (2021). Lateral hypothalamic LEPR neurons drive appetitive but not consummatory behaviors. *Cell Rep.* 36, 109615. doi: 10.1016/j.celrep.2021.109615

Singh, M. B., Babigian, C. J., and Sartor, G. C. (2022). Domain-selective BET inhibition attenuates transcriptional and behavioral responses to cocaine. *Neuropharmacology* 210, 109040. doi: 10.1016/j.neuropharm.2022.109040

Singh, P. K., and Lutfy, K. (2021). The role of beta-endorphin in cocaine-induced conditioned place preference, its extinction, and reinstatement in male and female mice. *Front. Behav. Neurosci.* 15, 763336. doi: 10.3389/fnbeh.2021.763336

Stojakovic, A., Ahmad, S. M., and Lutfy, K. (2021). Alterations of amphetamine reward by prior nicotine and alcohol treatment: the role of age and dopamine. *Brain Sci.* 11, 420. doi: 10.3390/brainsci11040420

Su, H., Bai, J., Fan, Y., Sun, T., Du, Y., Li, Y., et al. (2022). The distinct roles of various neurotransmitters in modulating methamphetamine-induced conditioned place preference in relevant brain regions in mice. *Neuroreport* 33, 101-108. doi: 10.1097/WNR.0000000000001760

Sutton, L. P., Khalatyan, N., Savas, J. N., and Martemyanov, K. A. (2021). Striatal RGS7 regulates depression-related behaviors and stress-induced reinstatement of cocaine conditioned place preference. *eNeuro* 8, ENEURO.0365-20.2020. doi: 10.1523/ENEURO.0365-20.2020

Tan, X., Liu, X., Liu, E., Liu, M., Mu, S., Hang, Z., et al. (2022). Astrocyte-derived lactate/NADH alters methamphetamine-induced memory consolidation and retrieval by regulating neuronal synaptic plasticity in the dorsal hippocampus. *Brain Struct. Funct.* 227, 2681-2699. doi: 10.1007/s00429-022-02563-1

Turner, B. D., Smith, N. K., Manz, K. M., Chang, B. T., Delpire, E., Grueter, C. A., et al. (2021). Cannabinoid type 1 receptors in A2a neurons contribute to cocaine-environment association. *Psychopharmacology* 238, 1121-1131. doi: 10.1007/s00213-021-05759-1

Valenti, O., Zambon, A., and Boehm, S. (2021). Orchestration of dopamine neuron population activity in the ventral tegmental area by caffeine: comparison with amphetamine. *Int. J. Neuropsychopharmacol.* 24, 832-841. doi: 10.1093/ijnp/pyab049

Veisi, A., Khaleghzadeh-Ahangar, H., Fattahi, M., and Haghparast, A. (2023). The role of orexin-1 receptors within the hippocampal CA1 Area in the extinction and reinstatement of methamphetamine-seeking behaviors. *Neurochem. Res.* 48, 671-680. doi: 10.1007/s11064-022-03793-9

Visser, E., Matos, M. R., Mitrić, M. M., Kramvis, I., van der Loo, R. J., Mansvelder, H. D., et al. (2022). Extinction of cocaine memory depends on a feed-forward inhibition circuit within the medial prefrontal cortex. *Biol. Psychiatry* 91, 1029-1038. doi: 10.1016/j.biopsych.2021.08.008

Wang, Z. Y., Guo, L. K., Han, X., Song, R., Dong, G. M., Ma, C. M., et al. (2021). Naltrexone attenuates methamphetamine-induced behavioral sensitization and conditioned place preference in mice. *Behav. Brain Res.* 399, 112971. doi: 10.1016/j.bbr.2020.112971

Wang, Y., Zhang, J., Deji, C., Fan, J., Miao, X., Li, S., et al. (2021). Differential perturbations of gut microbial profiles and co-occurrence networks among phases of methamphetamine-induced conditioned place preference. *J. Neurosci. Res.* 99, 2860-2873. doi: 10.1002/jnr.24963

Wang, Q., Guo, X., Yue, Q., Zhu, S., Guo, L., Li, G., et al. (2023a). Exploring the role and mechanism of gut microbiota in methamphetamine addiction using antibiotic treatment followed by fecal microbiota transplantation. *Anat. Rec.* 306, 1149-1164. doi: 10.1002/ar.25055

Wang, Y., Yang, L., Zhou, H., Zhang, K., and Zhao, M. (2023b). Identification of miRNA-mediated gene regulatory networks in L-methionine exposure counteracts cocaine-conditioned place preference in mice. *Front. Genet.* 13, 1076156. doi: 10.3389/fgene.2022.1076156

Weitz, M., Khayat, A., and Yaka, R. (2021). GABAergic projections to the ventral tegmental area govern cocaine-conditioned reward. *Addict. Biol.* 2021 26, e13026. doi: 10.1111/adb.13026

Wiah, S., Roper, A., Zhao, P., Shekarabi, A., Watson, M. N., Farkas, D. J., et al. (2023). Troriluzole inhibits methamphetamine place preference in rats and normalizes methamphetamine-evoked glutamate carboxypeptidase II (GCPII) protein levels in the mesolimbic pathway. *Drug Alcohol Depend.* 242, 109719. doi: 10.1016/j.drugalcdep.2022.109719

Xu, W., He, Y., Zhang, J., Li, H., Wan, X., Li, M., et al. (2021). Simvastatin blocks reinstatement of cocaine-induced conditioned place preference in male mice with brain lipidome remodeling. *Neurosci. Bull.* 37, 1683-1702. doi: 10.1007/s12264-021-00771-z

Xue, A., Huang, Y., Li, M., Wei, Q., and Bu, Q. Comprehensive analysis of differential m6a RNA methylomes in the hippocampus of cocaine-conditioned mice. *Mol. Neurobiol.* 58, 3759-3768. doi: 10.1007/s12035-021-02363-4

Yan, P. J., Ren, Z. X., Shi, Z. F., Wan, C. L., Han, C. J., Zhu, L. S., et al. (2022). Dysregulation of iron homeostasis and methamphetamine reward behaviors in Clk1-deficient mice. *Acta Pharmacol. Sin.* 43, 1686-1698. doi: 10.1038/s41401-021-00806-1

Yang, C., Fu, X., Hao, W., Xiang, X., Liu, T., Yang, B. Z., et al. (2021). Gut dysbiosis associated with the rats' responses in methamphetamine-induced conditioned place preference. *Addict. Biol.* 26, e12975. doi: 10.1111/adb.12975

Yang, G., Li, J., Peng, Y., Shen, B., Li, Y., Liu, L., et al. (2022). Ginsenoside Rb1 attenuates methamphetamine (METH)-induced neurotoxicity through the NR2B/ERK/CREB/BDNF signalings *in vitro* and *in vivo* models. *J. Ginseng Res.* 46, 426-434. doi: 10.1016/j.jgr.2021.07.005

Yates, J. R., Campbell, H. L., Hawley, L. L., Horchar, M. J., Kappesser, J. L., and Wright, M. R. (2021a). Effects of the GluN2B-selective antagonist Ro 63-1908 on acquisition and expression of methamphetamine conditioned place preference in male and female rats. *Drug Alcohol Depend.* 225, 108785. doi: 10.1016/j.drugalcdep.2021.108785

Yates, J. R., Horchar, M. J., Kappesser, J. L., Broderick, M. R., Ellis, A. L., and Wright, M. R. (2021b). The association between risky decision making and cocaine conditioned place preference is moderated by sex. *Drug Alcohol Depend.* 228, 109079. doi: 10.1016/j.drugalcdep.2021.109079

Yunusoğlu, O. (2021). Linalool attenuates acquisition and reinstatement and accelerates the extinction of nicotine-induced conditioned place preference in male mice. *Am. J. Drug Alcohol Abuse* 47, 422-432. doi: 10.1080/00952990.2021.1898627

Zhang, K., Ji, G., Zhao, M., and Wang, Y. (2021). Candidate l-methionine target piRNA regulatory networks analysis response to cocaine-conditioned place preference in mice. *Brain Behav.* 11, e2272. doi: 10.1002/brb3.2272

Zhang, L., Meng, S., Chen, W., Chen, Y., Huang, E., Zhang, G., et al. (2021). High-frequency deep brain stimulation of the substantia nigra pars reticulata facilitates extinction and prevents reinstatement of methamphetamine-induced conditioned place preference. *Front. Pharmacol.* 12, 705813. doi: 10.3389/fphar.2021.705813

Zhao, Z. D., Han, X., Chen, R., Liu, Y., Bhattacherjee, A., Chen, W., et al. (2022). A molecularly defined D1 medium spiny neuron subtype negatively regulates cocaine addiction. *Sci. Adv.* 8, eabn3552. doi: 10.1126/sciadv.abn3552

Zhou, Y., Xiao, S., Li, C., Chen, Z., Zhu, C., Zhou, Q., Ou, J., et al. (2021). Extracellular vesicle-encapsulated mir-183-5p from rhynchophylline-treated H9c2 cells protect against methamphetamine-induced dependence in mouse brain by targeting NRG1. *Evid. Based Complement. Alternat. Med.* 2021, 2136076. doi: 10.1155/2021/2136076

Zhu, C., Hong, T., Li, H., Jiang, S., Guo, B., Wang, L., et al. (2021a). Glucagon-like peptide-1 agonist exendin-4 facilitates the extinction of cocaine-induced condition place preference. *Front. Syst. Neurosci.* 15, 711750. doi: 10.3389/fnsys.2021.711750

Zhu, C., Tao, H., Rong, S., Xiao, L., Li, X., Jiang, S., et al. (2021b). Glucagon-like peptide-1 analog exendin-4 ameliorates cocaine-mediated behavior by inhibiting toll-like receptor 4 signaling in mice. *Front. Pharmacol.* 12, 694476. doi: 10.3389/fphar.2021.694476

Zhu, C., Wang, L., Ding, J., Li, H., Wan, D., Sun, Y., et al. (2022). Effects of glucagon-like peptide-1 receptor agonist exendin-4 on the reinstatement of cocaine-mediated conditioned place preference in mice. *Front. Behav. Neurosci.* 15, 769664. doi: 10.3389/fnbeh.2021.769664

Zhu, J., Quizon, P. M., Wang, Y., Adeniran, C. A., Strauss, M. J., Jiménez-Torres, A. C., et al. (2022). SRI-32743, a novel allosteric modulator, attenuates HIV-1 Tat protein-induced inhibition of the dopamine transporter and alleviates the potentiation of cocaine reward in HIV-1 Tat transgenic mice. *Neuropharmacology* 220, 109239. doi: 10.1016/j.neuropharm.2022.109239

Zhu, L., Wu, F., Yan, Z., He, L., Wang, S., Hu, H., et al. (2022). A novel microRNA, novel-m009C, regulates methamphetamine rewarding effects. *Mol. Psychiatry* 27, 3885-3897. doi: 10.1038/s41380-022-01651-2

Zipperly, M. E., Sultan, F. A., Graham, G. E., Brane, A. C., Simpkins, N. A., Carullo, N. V. N., et al. (2021). Regulation of dopamine-dependent transcription and cocaine action by Gadd45b. *Neuropsychopharmacology* 46, 709-720. doi: 10.1038/s41386-020-00828-z
